# Prussian‐Blue Catalysis and NFC Synergy: a Battery‐Free Laser‐Induced Graphene‐Based Platform for Urine Glucose Monitoring at Point‐of‐Care

**DOI:** 10.1002/advs.202500365

**Published:** 2025-03-06

**Authors:** Sinan Uzunçar, Gabriel Maroli, Massimo Urban, Arben Merkoçi

**Affiliations:** ^1^ Catalan Institute of Nanoscience and Nanotechnology (ICN2) CSIC and BIST Campus UAB, Bellaterra Barcelona 08193 Spain; ^2^ Environmental Engineering Department Engineering Faculty Zonguldak Bülent Ecevit University Zonguldak 67100 Turkey; ^3^ Instituto de Investigaciones en Ingeniería Eléctrica Alfredo Desages (IIIE) Universidad Nacional del Sur Bahía Blanca 8000 Argentina; ^4^ Facultad de Biociencias Universitat Autònoma de Barcelona Campus de la UAB, Bellaterra Barcelona 08193 Spain; ^5^ Catalan Institution for Research and Advanced Studies (ICREA) Passeig de Lluís Companys, 23 Barcelona 08010 Spain

**Keywords:** battery‐free, laser‐induced graphene, NFC, prussian‐blue, urine glucose monitoring

## Abstract

Prussian‐blue nanoparticles (PBNPs) show promise in electrochemical hydrogen peroxide (H_2_O_2_) sensing but face operational stability challenges without complex strategies. This study introduces a simplified, polymer‐based synthesis method, enhancing their stability in a single step. Chemical polymerization of Prussian‐blue (PB) and poly(3,4‐ethylenedioxythiophene) (PEDOT) with gelatin as a polycationic soft template yields a self‐assembled PB‐infused Catalytic Hetero‐interface Architecture (PB‐CHIA) that remarkably improves the stability of PBNPs and offers functional groups for enzyme immobilization, supporting robust biosensing applications. The softened PEDOT rigidity extends PB‐CHIA's applicability to various carbonaceous electrode substrates, including glassy carbon and laser‐induced graphene (LIG) via simple drop‐casting. A fluidic cell module designed with the optimized LIG morphology (nano‐fibrous fringes, LIG‐F, diameter: 72.87 ± 12.24 nm) modified with PB‐CHIA and glucose oxidase enables non‐invasive urine glucose monitoring. The configuration accurately quantifies glucose within a linear range of 10–400 µM [R^2^: 0.991, Sensitivity: 29.88 ± 4.98 µA mM⁻¹ cm⁻^2^, Detection Limit: 4.52 ± 2.24 µM], covering medical needs. A near‐field communication potentiostat is devised for a fully integrated, batteryless, wireless point‐of‐care (POC) prototype, enabling rapid smartphone readouts in 15 s for daily home‐based use. The stable operation of PB‐CHIA allows working electrodes’ scalable production, highlighting its potential for diverse POC devices in urinary analysis reliant on H_2_O_2_ assays.

## Graphical abstract

In this study, Merkoçi and his research group present a polymer‐based synthesis to enhance the stability of Prussian blue (PB) nanoparticles. The resulting PB‐infused catalytic hetero‐interface architecture (PB‐CHIA) achieves complete two‐dimensional (2D) and three‐dimensional (3D) substrate coverage (glassy carbon and laser‐induced graphene electrodes). PB‐CHIA enables enzyme immobilization for hydrogen peroxide (H2O2)‐dependent assays, yielding a wireless, battery‐free urine glucose sensor prototype.

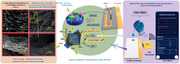

## 1 Introduction

*Diabetes mellitus* poses a significant and escalating health challenge. Individuals plagued with this condition are required to monitor their blood glucose levels throughout the day frequently, yet conventional blood sampling techniques present difficulties, particularly for those with phobias or underlying medical conditions.^[^ ^1^, ^2^ ^]^ Hence, non‐invasive approaches, such as urine glucose sensing, are emerging as viable alternatives, which not only play an active role in the management of diabetes but also provide valuable diagnostic perspicuity into conditions such as hyperthyroidism, kidney malfunction, and rare genetic disorders.^[^ ^3^, ^4^, ^5^, ^6^, ^7^ ^]^ Although electrochemical sensors show promising performance for urine glucose detection,^[^ ^8^ ^]^ there is limited focus on point‐of‐care (POC) devices for home disease management. Some studies have investigated wearable options like sensor‐equipped diapers,^[^ ^9^, ^10^, ^11^ ^]^ and others have developed portable POCs for self‐monitoring urine glucose (SMUG) without age or illness‐related restrictions on sample collection.^[^ ^12^, ^13^, ^14^ ^]^ Developing POCs for SMUG could be advantageous as it might serve a broader range of demographics, for which an inexpensive, battery‐free device would be mandatory.

Researchers have extensively explored catalytic nanomaterials as artificial peroxidases in biosensor development. These innovative materials, composed of carbon, metals, or their combinations, are designed to detect a range of substances, including choline, cholesterol, lactic acid, and glucose. In these biosensors, specific oxidase enzymes target each analyte, producing H_2_O_2_ as a by‐product. The catalytic nanomaterials then generate signals by reducing or oxidizing this H_2_O_2_.^[^ ^15^ ^]^ Among them,^[^ ^16^ ^]^ Prussian Blue (PB), a hexacyanoferrate mixed‐valence 3D‐coordination compound Fe_4_^III^[Fe^II^(CN)_6_]_3_, leaps out due to its simplicity of production, cost‐effectiveness, and superior sensitivity and selectivity toward H_2_O_2_.^[^ ^16^, ^17^, ^18^, ^19^, ^20^, ^21^, ^22^, ^23^ ^]^ Reductive detection of H_2_O_2_ at a low applied potential (≈0.0 V vs Ag/AgCl) is advantageous because it minimizes interference from common electrochemical species.^[^ ^24^ ^]^ However, the effectiveness of PB‐based H_2_O_2_ sensing is limited by the instability of the Fe‐CN‐Fe bonds in the PB unit cell, which are compromised by the OH^−^ released during the electrochemical reduction of H_2_O_2_. This phenomenon results in material loss and decreased electrochemical activity of the glucose‐sensitive interface during its operation, creating a severe handicap in designing sensors reliant on PBNPs.^[^ ^25^ ^]^

Researchers have spent the last two decades exploring various methods aimed at enhancing the stability of PBNPs in sensor applications. These methods contain a wide range of strategies, from the optimization of synthesis procedures^[^ ^25^, ^26^ ^]^ to the enhancement of PB adsorption or binding on carbon‐based electrodes through functional group incorporation^[^ ^27^, ^28^, ^29^ ^]^ and employment of various encapsulation techniques, such as metal‐enriched polymeric membranes^[^ ^30^ ^]^ and metal‐based electrode arrays.^[^ ^31^ ^]^ Additionally, entrapping PBNPs with Ni‐hexacyanoferrate isostructural coverages,^[^ ^32^, ^33^ ^]^ implementing non‐conductive layers,^[^ ^34^, ^35^ ^]^ and incorporating surfactants^[^ ^36^ ^]^ have been investigated. Despite significant advancements in this area, these methods often constitute complex and multi‐stage processes. Another effective strategy is the incorporation of the conductive polymers into the PB‐based interface. These polymers, with their flexible chain structure, provide modulable electrical and optical properties,^[^ ^37^, ^38^ ^]^ enhancing the stability of sensor electrodes compared to using pristine PB alone.^[^ ^39^ ^]^ However, for water‐insoluble monomers, there is a need to use solvents that are harmful to the environment and human health, such as acetonitrile,^[^ ^40^ ^]^ dimethylsulfoxide (DMSO)^[^ ^41^ ^]^ or dichloromethane.^[^ ^42^ ^]^ To avoid the use of these solvents, the immobilization of the pre‐synthesized PBNPs inside hetero‐interfaces through the electrochemical polymerization of conductive polymers aided by soft templates has also been explored. This method significantly increases the operational stability of PBNPs, enhancing biosensor robustness by providing functional groups from the soft templates for enzyme immobilization.^[^ ^43^, ^44^, ^45^, ^46^ ^]^ However, challenges arise when implementing these hetero‐interfaces in practical biosensing devices with three‐electrode systems on a single substrate. The process, which relies on electrochemical polymerization, inevitably cross‐modifies the reference and counter electrodes, requiring additional fabrication procedures.

This study suggests the construction of a PB‐based hetero‐interface on electrode surfaces through chemical polymerization rather than electrochemical polymerization. This aims to tackle PB's operational stability issue and streamline biosensing device fabrication with three‐electrode systems on a single substrate. We controlled the synthesis of PBNPs by simultaneously infusing PB's precursors with both the soft template and the conductive polymer monomer. Herein, as a conductive polymer, we utilize poly(3,4‐ethylenedioxythiophene) (PEDOT) for its superior capacitance and conductivity compared to others.^[^ ^47^, ^48^ ^]^ Additionally, we select gelatin (G) as a polycationic soft template for dispersing PEDOT in water to eliminate the use of solvents. This strategic combination leads to our innovative approach of architecting a hetero‐interface, where the self‐assembling, intertwined structure of PB‐PEDOT‐gelatin gradually evolves into an interconnected PB‐infused Catalytic Hetero‐interface Architecture (PB‐CHIA) during the polymerization process. This evolution results in a physical blockage layer that enhances the operational stability of PBNPs. Moreover, the presence of gelatin supplies functional groups for the immobilization of enzymes.

**Figure 1:**
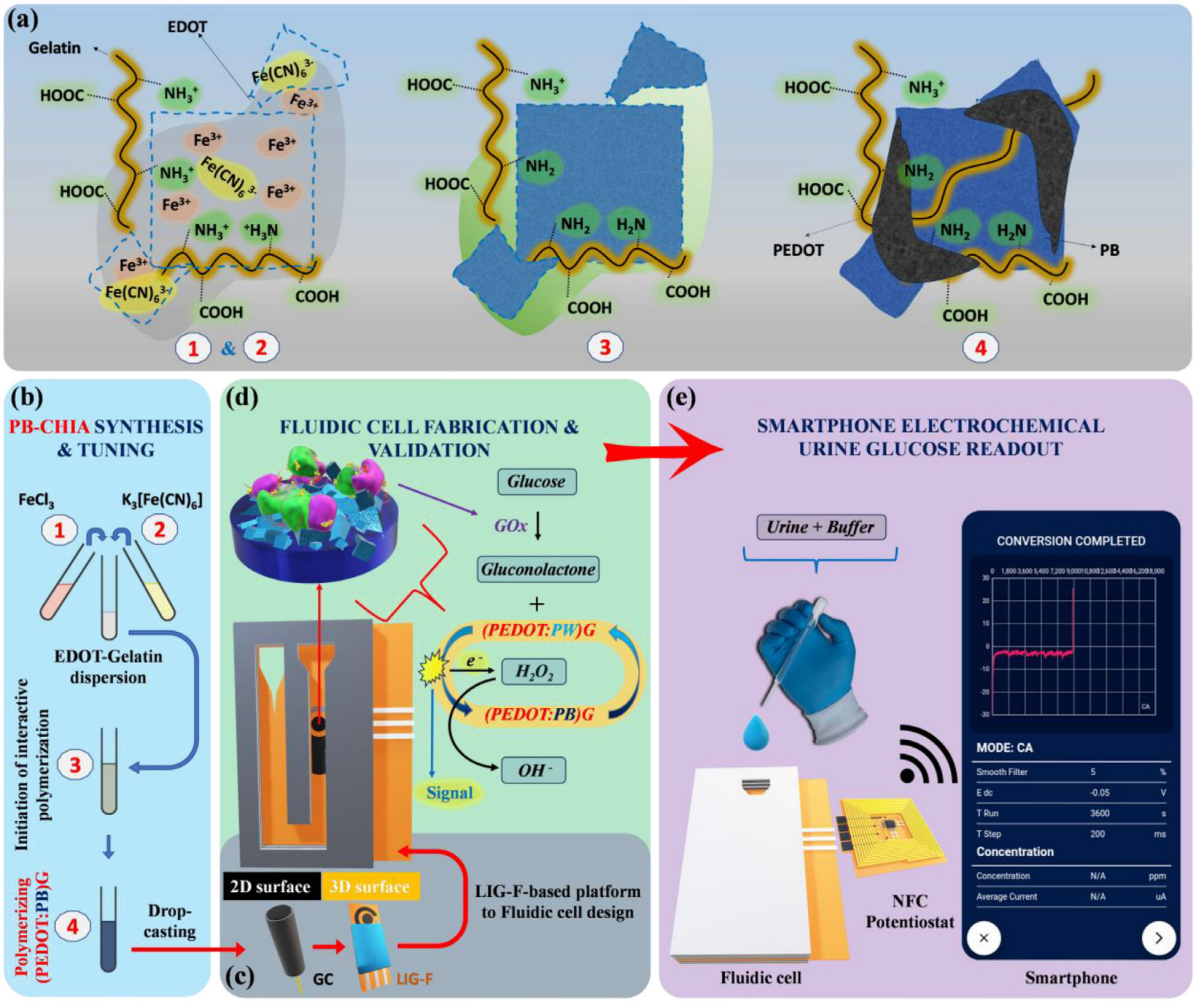
Roadmap of stable PB‐CHIA synthesis to a hydrophilic flow‐driven, portable, battery‐free, and wireless fluidic cell prototype for smartphone electrochemical urine glucose readouts: a, b) Synthesis of PB‐CHIA Composite: Sequential addition of EDOT, FeCl_3_, and K_3_[Fe(CN)_6_] to a water‐based solution containing HCl, KCl, and gelatin yields PB‐CHIA. Gelatin acts as a polycationic soft template, dispersing EDOT and providing nucleation sites for PBNPs. FeCl_3_ initiates and dopes the polymerization of 3,4‐Ethylenedioxythiophene (EDOT). The addition of K_3_[Fe(CN)_6_] induces PBNP formation, with nanoparticles coordinating with free Fe^3+^ cations, those involved in PEDOT doping, and previously bound to gelatin's protonated ‐NH_2_ groups, leading to the self‐assembly of an intertwined (PEDOT:PB)G architecture. The resulting composite, nicked by gelatin, enhances operational stability and provides functional groups for glutaraldehyde cross‐linking. Additionally, cross‐linking with the GOx enzyme further improves the stability of PBNPs in PB‐CHIA. c) Application of PB‐CHIA to Electrode Surfaces: Optimized precursor addition ensures effective polymerization on both glassy carbon (GC) electrodes (2D surface) and nano‐textured LIG electrodes (3D surface). This method facilitates POC device fabrication using printed LIG platforms while safeguarding reference and counter electrodes. d) Design of LIG‐Based Fluidic Cell Module: Utilizing PB‐CHIA on LIG surfaces with nano‐fibrous fringes improves electroanalytical performance compared to GC substrates. The nano‐fibrous fringes act as nanoelectrodes, enhancing charge transfer at the solid‐liquid junction by increasing film porosity and contact surface. A hydrophilic flow‐driven fluidic cell module, designed with LIG on a polyimide substrate, modified with PB‐CHIA, and cross‐linked with GOx enzyme, detects glucose through H_2_O_2_. The module supports multiple sample injections and analyte quantification. e) NFC‐Enabled Wireless Glucose Sensing Prototype: Integration of a custom‐designed NFC system into the fluidic cell module enables wireless, battery‐free operation via smartphones, highlighting the device's POC potential for SMUG and versatility in biosensing based on H_2_O_2_ assay.

**Figure** **1** illustrates the roadmap of the present work in this direction. Starting with the synthesis and application strategy of PB‐CHIA (Figure 1a,b) and comparing its performance on both glassy carbon (GC) electrodes, offering a 2D surface area, and LIG‐based electrode surfaces, which provide a 3D surface area (Figure 1c) and are popular for their versatility in POC device fabrication.^[^ ^49^, ^50^ ^]^ Our synthesis and application approach for PB‐CHIA supports the mass production of POCs reliant on LIG platforms since the synthesis can be directly conducted on working electrode surfaces using the polymerization medium, thus protecting the reference and counter electrodes. Toward the end of the study, we developed a PB‐CHIA‐modified laser‐induced graphene (LIG) on a polyimide‐based fluidic cell module, which enabled the detection of glucose from multiple samples (Figure 1d). The system allows continuous monitoring through a reusable NFC module. The fluidic cell can be replaced at the end of its lifespan. Through the integration of these components and the immobilization of the glucose oxidase (GOx) enzyme on PB‐CHIA, we demonstrate the functionality of the innovative, fully integrated, wireless, battery‐free urine glucose smartphone readout prototype (Figure 1e). The suggested POC for SMUG applications using synthetic urine can be validated in real‐life scenarios. It is thought that the PB‐CHIA synthesized in the study can be modified with other oxidase‐based enzymes, such as cholesterol oxidase, choline oxidase, and lactate oxidase, depending on the H_2_O_2_ assay. Thus, various POC devices can be obtained by changing the enzyme used in the proposed prototype, significantly contributing to urine analysis at home.

## 2 Results and Discussion

First, our study will focus on the synthesis, optimization, and characterization of PB‐CHIA in the forthcoming sections. We will perform these steps on the 2D GC electrode surface and then investigate the applicability of the optimized PB‐CHIA on a 3D nano‐textured surface provided by the LIG on the polyimide substrate. In detail, we will compare the electrochemical performance of the working electrode surfaces in both 2D and 3D configurations. Afterward, we will design a fluidic cell module based on LIG on a polyimide, allowing us to introduce multiple samples and calibrate the sensor system. Finally, we will integrate the custom‐designed near‐field communication (NFC) module into the fluidic cell module to demonstrate the practicality of the yielding batteryless, wireless prototype as a POC device for the SMUG application using a smartphone.

### 2.1 (PEDOT:PB)G‐Based PB‐CHIA for the Prototype

#### 2.1.1 Synthesis, Chemical, and Physical Characterization of PB‐CHIA

The precipitation reaction between FeCl_3_ and K_3_[Fe(CN)_6_] yields a yellow‐green‐colored ferrous ferrocyanide salt in water. Upon the interaction of the precipitate with a reducing agent like H_2_O_2_ or certain organic materials such as polymers or oxidizable monomers, the color switches to blue, which indicates a change in the size distribution of PBNPs, which also determines their electrocatalytic activity toward H_2_O_2_.^[^ ^51^ ^]^ In our synthesis process, we modulate the electroactivity of PB through PEDOT polymerization and its interaction with functional groups exists in gelatin. This results in a structure with extended electrochemical stability of PB and is capable of immobilizing enzymes for biosensor production. Using this material, the mass production of the POC devices can be simplified.

The synthesis process of PB‐CHIA involves chemical polymerization by the sequential addition of 3,4‐Ethylenedioxythiophene (EDOT), FeCl_3_, and K_3_[Fe(CN)_6_] to a water‐based solution containing HCl, KCl, and gelatin (Figure 1a,b). If the last precursor of PB, K_3_[Fe(CN)_6_], is added before FeCl_3_, the lag phase, which is useful for the transportation of the polymerization mixture to a substrate surface, cannot be ensured in the polymerization reaction and uncontrolled product formation occurs. Therefore, the order of inputs mentioned earlier helps control reaction kinetics in the lag phase and optimize the formation of the desired product by varying the PB's precursor concentrations. Acting as a polycationic soft template under acidic conditions, gelatin disperses EDOT in water and provides a uniform medium for polymerization. The introduction of FeCl_3_ initiates EDOT polymerization and simultaneously dopes the growing PEDOT chains to maintain electroneutrality.^[^ ^52^, ^53^, ^54^ ^]^ During PEDOT polymerization, Fe^3+^ ions interact with protonated ‐NH_2_ groups (‐NH_3_^+^) in gelatin, which are acting as Lewis bases. This interaction leads to the formation of coordinate bonds with Fe^3+^ ions by converting ‐NH_3_^+^ to an uncharged, ‐NH_2_ form by releasing a proton.^[^ ^55^ ^]^

Upon adding K_3_[Fe(CN)_6_], the mixture undergoes a rapid color change to dark blue, which indicates the production of PBNPs. Formation of these nanoparticles occurs through the coordination of Fe(CN)_6_^3−^ anions with Fe^3+^ cations, which previously coordinated with ‐NH_3_^+^ groups in gelatin, as well as with free Fe^3+^ ions and those on the progress of doping the polymerization of PEDOT. This contributes to the overall electroneutrality of the structure. Infusion of Fe(CN)_6_^3−^ and Fe^3+^ into the PEDOT and gelatin chains triggers an interactive polymerization process between PEDOT and PB that results in a self‐assembling intertwined structure of PEDOT:PB. This structure, further nicked by gelatin, gradually evolves into an interconnected composite (PEDOT:PB)G, which forms a physical blockage layer covering the generated PBNPs that improves its operational stability. The gelatin in (PEDOT:PB)G structure also supplies hydroxyl groups (–OH, from hydroxyproline, hydroxylysine, and serine) in addition to amino groups (‐NH_2_, from lysine and hydroxylysine). These functional groups serve as sites for glutaraldehyde to create hydrophobic pockets for PB by allowing the cross‐linking reactions between gelatin chains and GOx.^[^ ^56^, ^57^ ^]^ This entity ensures an additional improvement in PB's physical and electrochemical stability within PB‐CHIA.

**Figure 2:**
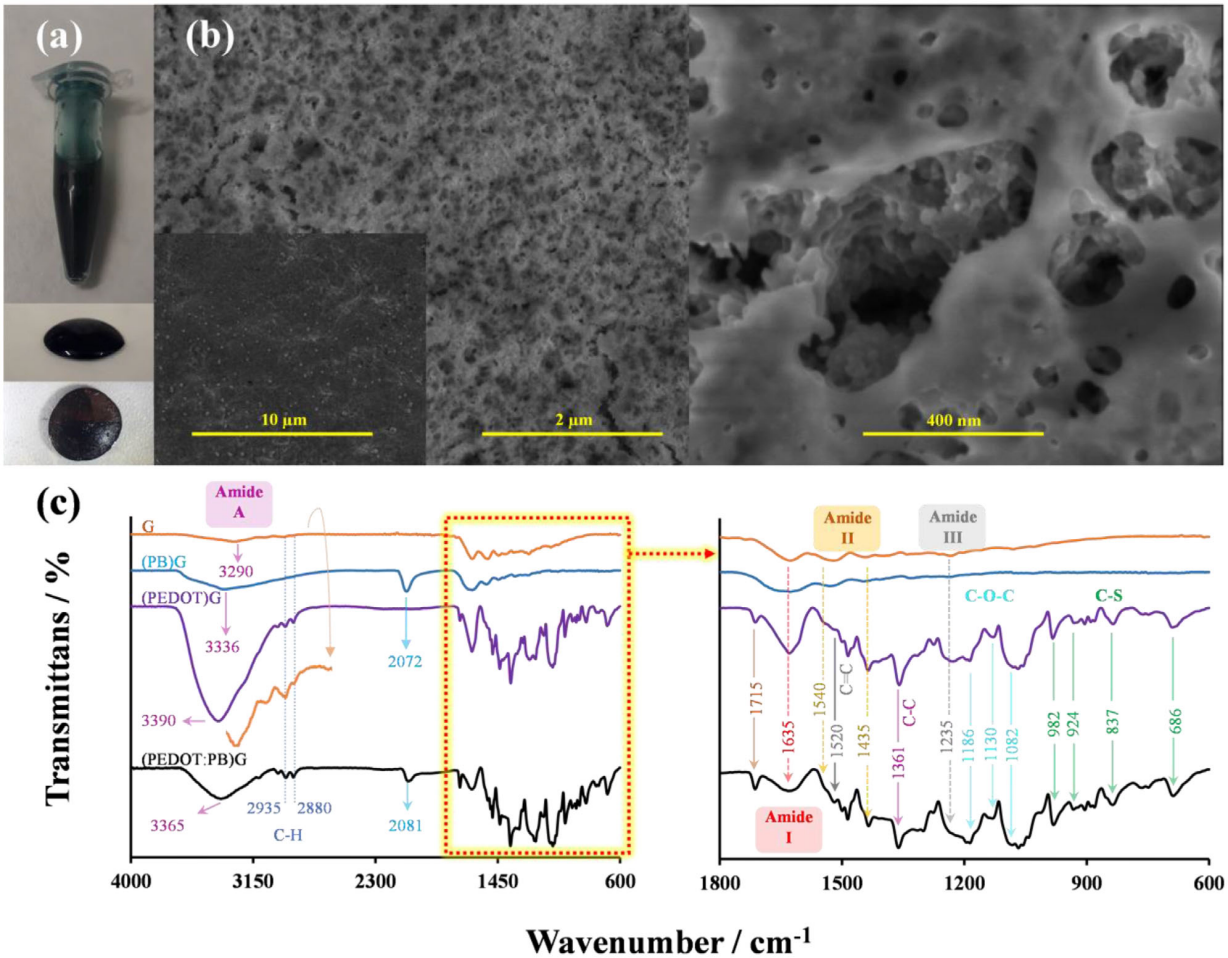
PB‐CHIA synthesis and characterization. a) Polymerization medium, droplet, and dried film, b) SEM images progress from lower to higher magnification of (PEDOT:PB)G film from the inset to the right. c) FTIR spectra of building blocks and resulting (PEDOT:PB)G.

The resulting mixture from interactive polymerization forms a growing (PEDOT:PB)G structure water dispersion (**Figure** **2**a; Figure S1a, Supporting Information), suitable for surface modification through drop‐casting. After dispersion is deposited onto a substrate surface, it gradually dries, forming a porous film that adheres firmly to the surface as polymerization proceeds. In contrast to the flat film offered by PB‐free (PEDOT)G, in situ generation of PB and its infusion yield a rough matrix of (PEDOT:PB)G (Figure S1b, Supporting Information) with an estimated pore size ranging between 10 and 400 nm (Figure 2b), as seen in SEM images.

As shown in Figure 2c the FT‐IR was used to characterize the molecular structure of (PEDOT:PB)G, which consists of gelatin, PB, and PEDOT. Noteworthy bands of gelatin's amide A at 3290 cm⁻¹ indicate N‐H stretching vibrations, while bands at 2935 and 2880 cm⁻¹ in the G spectrum are associated with C‐H stretching vibrations. Amide I, II, and III bands are also observed at 1635, 1549, and 1240 cm⁻¹, respectively.^[^ ^58^, ^59^, ^60^ ^]^ Except for the amide A band, all the other bands mentioned are seen in all combinations containing gelatin, almost without changing their positions.

The amide A band of gelatin shifts to 3336 and 3390 cm⁻¹ when combined with PB [(PB)G spectra] and PEDOT [(PEDOT)G spectra]. After combining all the elements, the obtained (PEDOT:PB)G structure's amide A band is observed at 3365 cm⁻¹. Additionally, the PB's C≡N group stretching vibration band^[^ ^31^, ^55^ ^]^ observed for the G(PB) composite at 2072 cm⁻¹ slightly shifts to 2081 cm⁻¹ with the inclusion of PEDOT yielding (PEDOT:PB)G composite. These shifts, mainly observed in the amide A region, indicate the interaction between components within the heterostructure, supporting the proposed mechanism illustrated in Figure 1a. Again, as a result of the in‐situ polymerization, a sharp peak appears at 1715 cm^−1^ for (PEDOT)G and G(PEDOT:PB), which is attributed to the water bending likely remaining trapped inside during the intricate porous structure formation.^[^ ^61^ ^]^

On the other hand, due to the high atom count of the (PEDOT:PB)G, the lower portion of the spectra is crowded from the fingerprint region of the different parts of the composite. However, characteristic features such as the thiophene ring's vibrational modes (C = C at 1520 cm⁻¹, C‐C stretches at 1361 cm⁻¹, and C‐O‐C/C–S vibrations at 1186, 1130, 1082/982, 924, 837, and 686 cm⁻¹)^[^ ^62^, ^63^ ^]^ are apparent in the composites of (PEDOT)G and (PEDOT:PB)G. The observation of characteristic bands corresponding to all building blocks within the PB‐CHIA also indicates that the soft template polymerization process successfully converted them into the (PEDOT:PB)G structure. Figure S1 (Supporting Information) displays the color variations resulting from the interactions of different combinations of the components in composite production.

#### 2.1.2 Optimization and Electrochemical Characterization of PB‐CHIA

Throughout the study, H_2_O_2_‐sensitive electrodes were obtained by preparing a PB‐CHIA polymerization mixture, which was then applied onto GC and LIG working electrode surfaces, providing 2D and 3D surface morphologies. For the fabrication of biosensor electrodes, enzyme immobilization on the PB‐CHIA‐modified electrode surfaces was achieved by cross‐linking the surface with glutaraldehyde. Further details on the fabrication process can be found in the Supporting Information, Section 1.3–1.4 (Figure S2–S4, Supporting Information).

First, we optimized the H_2_O_2_‐sensitive PB‐CHIA (PEDOT:PB)G, investigating the electrolyte's polymerization conditions, such as acidity and the volume of PB's precursor. To achieve the highest sensitivity glucose biosensor, the PB‐CHIA thickness on the electrode surface was also adjusted by diluting the polymerization medium with the selected electrolyte before the immobilization step of the GOx enzyme. The forthcoming subsections discuss the synergism between PB‐PEDOT and gelatin and tuning the yielding PB‐CHIA for glucose detection. Following these optimization stages, we will examine the applicability of the LIG‐based electrode surface for the development of a glucose‐sensitive prototype.

#### 2.1.3 Operational Mechanism of H_2_O_2_ and Glucose Sensors

Cyclic voltammetry (CV) redox curves in phosphate‐buffered saline (0.05 M PBS + 0.1 M KCl, pH 6) solution exhibits an anodic PB (Fe_4_^III^[Fe^II^(CN)_6_]_3_) and cathodic Prussian‐white (PW, K_4_Fe_4_^II^[Fe^II^(CN)_6_]_3_) peak couple (Equation 1). During amperometric measurements, the application of a negative potential (−0.05 V) to the electrode modified with (PEDOT:PB)G converts PB to PW, thereby stabilizing amperometric blank currents Equation 1). For the H_2_O_2_‐sensitive electrodes, after adding an H_2_O_2_‐containing sample to the reaction medium, the PW is exposed to H_2_O_2_ and is oxidized back to PB form, while H_2_O_2_ undergoes reduction to OH^−^, consuming electrons (Equation 2). Operational thermodynamics favor the conversion of PB to PW (Equation 1). Throughout these reactions, the consumption of electrons produces measurable amperometric reduction currents for the detection of H_2_O_2_.

For the glucose biosensors, the process involves a similar mechanism. After adding a glucose‐containing sample to the reaction medium, the immobilized GOx on the (PEDOT:PW)G catalyzes the oxidation of glucose to produce gluconolactone and H_2_O_2_ (Equation 3). The produced H_2_O_2_ then participates in the same reaction chain as described for the H_2_O_2_ sensors (Equations 1 and 2), allowing the detection of glucose through H_2_O_2_.

(1) $$\left(\mathrm{PEDOT}:\mathrm{PB}\right)G+e^{-}↔\left(\mathrm{PEDOT}:\mathrm{PW}\right)G$$

(2) $$\left(\mathrm{PEDOT}:\mathrm{PW}\right)G+H_{2}O_{2}\to \left(\mathrm{PEDOT}:\mathrm{PB}\right)G+2HO^{-}$$

(3) $$\mathrm{Glucose}+O_{2}\overset{GOx}{\to }\mathrm{Gluconolactone}+H_{2}O_{2}$$

#### 2.1.4 Effect of Polymerization Acidity and Precursor Volume of PB

**Figure 3:**
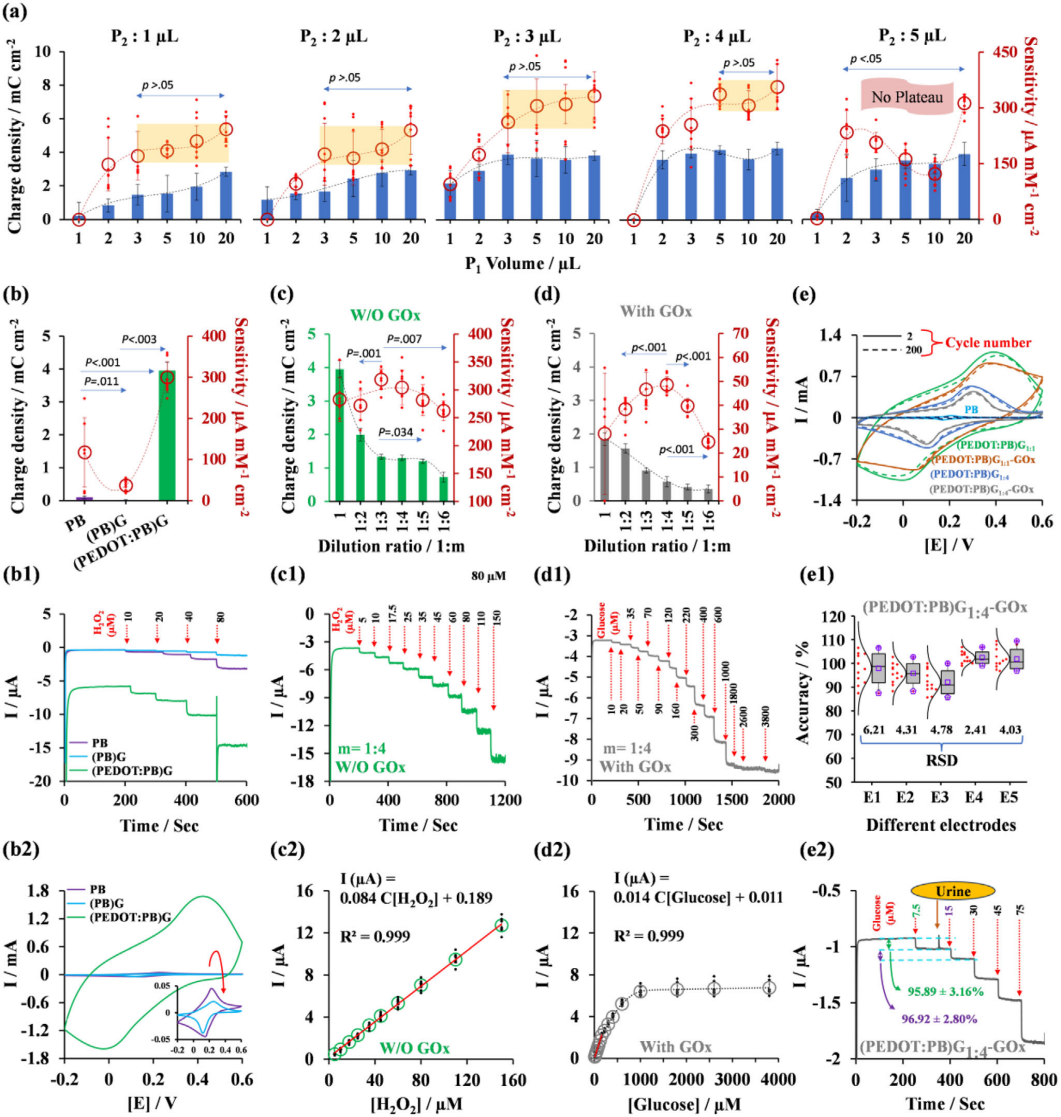
Optimization and Electrochemical Characterization of PB‐CHIA. a) Effects of varying P_1_ and P_2_ on charge density and sensitivity values. b) Synergistic effect on charge density and amperometric sensitivity arising from the combination of PB, PEDOT, and gelatin. b1) Amperometric signals toward H₂O₂ and b2) CV‐redox signals of stepwise modified electrodes, including GC/PB, GC/(PB)G, and GC(PEDOT:PB)G. c, d) Charge density and amperometric sensitivity value variations based on the film thickness adjusted by the dilution process, both without and with the GOx enzyme. c1–d1) Amperometric signals toward respective analytes and c2–d2) corresponding calibration plots with the optimal thickness obtained by an *n* = 1:4 dilution process. e Stability of CV‐redox signals for GC/(PEDOT:PB)G_1:1_ and GC/(PEDOT:PB)G_1:4_, both with and without GOx, and GC/PB in PBS. e1) Variation of five electrodes’ sensitivity toward 12‐time repetitive usage between 10–50 µM glucose. e2) Interference study showing anti‐interfering glucose responses in synthetic urine‐spiked PBS. (All CV‐redox signals were obtained at a scan rate of 0.05 V s^−1^ and amperometric measurements were performed at an applied potential of −0.05 V. Oppressional solution is PBS for both techniques. In the figures, column plots represent average charge density values (*n* = 3), while hollow circles indicate average sensitivity values (*n* = 9). Error bars show the standard deviation of these averages. Data points depict individual sensitivity measurements within each dataset. *p*‐values are calculated using one‐way ANOVA followed by post‐hoc Tukey's HSD tests. The linear response regions of the sensor to analyte additions (*n* = 9, where R^2^ > 0.990) are indicated by a solid red line).

The charge density values derived from the CV‐redox curves in PBS solution and the amperometric sensitivities toward H_2_O_2_ at the (PEDOT:PB)G interface were unaffected by the acidity of the polymerization electrolyte (Figure S5, Supporting Information). Therefore, consistent with the PB synthesis literature,^[^ ^25^, ^64^ ^]^ the most acidic polymerization medium (0.1 mm KCl + 0.1 mm HCl) was chosen to continue the study. Changes of the precursor‐1 (P_1_: FeCl_3_, 4 m) amount from 1 to 20 µL (1‐2, 3, 5, 10, and 20 µL) while varying the precursor‐2 (P_2_: K_3_[Fe(CN)_6_], 1 m) amount (from 1, 2, 3, 4, to 5 µL) in the polymerization medium led to a hyperbolic trend for amperometric response toward H_2_O_2_ and charge density values (**Figure** **3**a). For each P_2_ value, surpassing a certain P_1_ threshold led to a plateau in sensitivity, indicating the saturation of PB‐CHIA with PB. Post‐Hoc Tukey's HSD analysis (for each P_1_ and P_2_ trial, *n* = 9) indicated that when assessing all combinations of P_1_ values from high (20 µL) to low (1 µL) for each P_2_ value, the average sensitivity values for at least three distinct P_2_ conditions showed no significant differences (*p* > 0.05). Specifically, when P_2_ was set at 1, 2, or 3 µL, sensitivity values plateaued between P_1_ values of 3 and 20 µL. For P_2_ = 4, this plateau shifted, narrowing to a range between P_1_ values of 5 and 20 µL. In contrast, P_2_ values greater than 4 µL result in a distinct trend due to coagulates, disrupting polymerization dynamics. To ensure consistent polymerization, we standardized the volumes of P_1_ and P_2_ at 10 and 4 µL, respectively, which resulted in the optimal plateau for amperometric sensitivity and relatively high charge density.

#### 2.1.5 PB‐PEDOT‐Gelatin Synergism

The PB‐CHIA consists of three primary components, PEDOT, gelatin, and the only component that generates a well‐defined redox peak couple and amperometric signal for H_2_O_2_ additions, PB. Figure 3b compares the sensitivity and charge density values of PB, (PB)G and (PEDOT:PB)G (prepared via P_1_: 10 µL and P_2_: 4 µL) obtained from amperometric H_2_O_2_ measurement (Figure 3b1) and CV‐redox operation (Figure 3b2) in PBS using GC electrodes. According to the post‐hoc Tukey's HSD analysis of the mean sensitivity values, significant differences were observed among the three conditions (*p *< 0.05 for each electrode configuration, *n* = 9). GC/PB electrode presents charge density and amperometric sensitivity values of 0.102 mC cm^−2^ (E_PW_: 0.158 V, E_PB_:0.233 V, ∆E_PB‐PW_: 0.075 V) and 117 µA mM^−1^ cm^−2^, respectively. Synthesis of PB in gelatin causes 16.7% and 33.3% decrease in those values to be 0.017 mC cm^−2^ (E_PW_: 0.121 V, E_PB_: 0.261 V, ∆E_PB‐PW_: 0.140 V) and 39 µA mM^−1^ cm^−2^, respectively. Notably, the simultaneous infusion of PB into gelatin and PEDOT produces a synergistic effect by an increase of 3692.5% and 256.4% in charge density and sensitivity values compared to GC/PB, to be 3.951 mC cm^−2^ (E_PW_: 0.0132 V, E_PB_:0.426 V, ∆E_PB‐PW_: 0.413 V) and 300 µA mM^−1^ cm^−2^, respectively. This considerable change can be attributed to PEDOT's excellent conductivity and capacitive properties^[^ ^65^, ^66^ ^]^ that can be blended with gelatin, yielding a porous intertwined structure.

#### 2.1.6 Effect of PB‐CHIA Thickness and Quantification for Glucose

Electroactive film thickness on the electrode surface significantly affects sensor performance by affecting electrochemical reaction kinetics. Thicker films can increase diffusion paths and charge transfer resistance, reducing sensitivity. In contrast, thinner films improve mass transfer dynamics by decreasing diffusion paths and resistance but may lack sufficient electroactive material, compromising performance. In our study, while a thick PB‐CHIA film on GC was compelling for H₂O₂ sensing, it resulted in inconsistent glucose sensitivities, sometimes with no response. This inconsistency originated from diffusion barriers in the thicker PB‐CHIA layers, which hindered glucose and H₂O₂ transport to the electroactive interface, slowing reaction kinetics and reducing sensitivity.

To tackle these issues, we optimized the PB‐CHIA thickness by varying the dilution ratios of the polymerization medium with an electrolyte (0.1 M HCl + 0.1 M KCl), creating different thicknesses for GC/(PEDOT:PB)G_m_‐GOx electrodes. Control electrodes (GC/(PEDOT:PB)G_m_) without GOx were also prepared. The “m” notation in (PEDOT:PB)G_m_ denotes the volumetric ratio of pure polymerization medium (a) to the total volume (a+b) after adding electrolyte (b). For instance, a 1:4 dilution ratio reflecting (PEDOT:PB)G_1:4_ results from adding 150 µL of electrolyte to 50 µL of the main polymerization medium.

For H₂O₂ sensors, the 1:3 dilution ratio yielded the highest mean sensitivity at 318.74±46.66 µA mM⁻¹ cm⁻^2^, significantly outperforming 1:2 (272 µA mM⁻¹ cm⁻^2^, *p* < 0.001), 1:5 (281.7 µA mM⁻¹ cm⁻^2^, *p* = 0.034), and 1:6 (262.52 µA mM⁻¹ cm⁻^2^, *p* = 0.007). For glucose sensors, the 1:1 dilution ratio showed inconsistent results and was excluded from the analysis. Among them, the 1:4 dilution ratio provided the best mean sensitivity at 48.62 µA mM⁻¹ cm⁻^2^, significantly surpassing 1:2 (38.38 µA mM⁻¹ cm⁻^2^, *p *< 0.001), 1:3 (42.05 µA mM⁻¹ cm⁻^2^, *p* < 0.001), 1:5 (39.75 µA mM⁻¹ cm⁻^2^, *p* < 0.001), and 1:6 (24.69 µA mM⁻¹ cm⁻^2^, *p* < 0.001).

The optimal dilution ratios seem to be 1:3 for H₂O₂ and 1:4 for glucose sensors, statistically. Due to this difference, we additionally compared the sensitivity ratios (*S*_*H*2*O*2_/*S_Glucose_*), which mirrors the efficiency of translating the H₂O₂‐sensitive interface to glucose sensing. Ideally, these sensitivities should match since 1 mole of glucose is oxidized to 1 mole of H₂O₂ (Equation 3). A ratio close to 1 indicates better efficiency. However, variations are expected due to the crosslinking agent (glutaraldehyde), which can alter the PB‐CHIA layer's chemistry and enzyme conformation. Among the observed ratios (*S*_*H*2*O*2_/*S_Glucose_* = 10.07, 7.08, 6.83, 6.26, 7.08, and 10.63), the 1:4 dilution ratio yielded the closest ratio of 6.26 to 1, demonstrating the most effective conversion.

In the linear range with R^2^ > 0.990, the lowest sensitivity ratio *S*_*H*2*O*2_/*S_Glucose_* for n = 1:4) corresponded to the highest glucose sensitivity (48.62 ± 3.42 µA mM⁻¹ cm⁻^2^ with the enzyme‐modified GC/(PEDOT:PB)G_1:4_ electrode), which also exhibited a H₂O₂ sensitivity of 298.71 ± 8.90 µA mM⁻¹ cm⁻^2^. The glucose sensor had a linear response within 10–160 µM, described by the equation “I(µA) = 0.014C_[Glucose]_+0.011” (Figure 3d1,d2) (R^2^ = 0.999), with a Limit of Detection (LOD) of 1.16±0.47 µM. The H₂O₂ sensor showed a LOD of 0.33 ± 0.08 µM in the same range, following the equation “I [(µA) = 0.084C_[H₂O₂]_ +0.189” (Figure 3c1,c2) (R^2^ = 0.999). These results prompt further examination of specific electroanalytical characteristics, such as operational stability, precision, and applicability in urine‐spiked media for the GC/(PEDOT:PB)G_1:4_‐GOx sensor, which will be detailed in the following section.

#### 2.1.7 Operational Stability, Precision and Applicability in Urine‐Spiked Medium

The previous section noted that the GC/(PEDOT:PB)G_1:4_‐GOx electrode performed the highest amperometric sensitivity to glucose. Consequently, we initiated the electrochemical characterization by evaluating its operational cycling stability through 200 voltammetric cycles within a potential range of −0.2/+0.6 V at a scan rate of 0.05 V s^−1^ (Figure 3e). For comparison, GC/(PEDOT:PB)G_1:4_, GC/(PEDOT:PB)G_1:1_, GC/(PEDOT:PB)G_1:1_‐GOx and GC/PB electrodes were also prepared. In cases without enzyme immobilization, the interfaces on the GC electrodes were not cross‐linked with glutaraldehyde.

Charge density losses after 200 cycles were 13.81% and 9.26% for GC/(PEDOT:PB)G_1:1_ and GC/(PEDOT:PB)G_1:4_, respectively. On the other hand, for the GOx‐immobilized electrodes, GC/(PEDOT:PB)G_1:1_‐GOx and GC/(PEDOT:PB)G_1:4_‐GOx, the decreases were relatively lower, at 3.02% and 8.62%, respectively. Meanwhile, the GC/PB control electrode showed a charge density loss of 42.70%. This observation suggests that creating hydrophobic pockets for PBNPs during enzyme immobilization is crucial for enhanced operational cycling stability of PBNPs. Therefore, GC/(PEDOT:PB)G_1:1_‐GOx with a higher amount of gelatin showed the highest operational potential cycling stability compared to its diluted version (GC/(PEDOT:PB)G_1:4_‐GOx). However, the 1:4 dilution ratio of PB‐CHIA proved optimal for achieving the highest amperometric sensitivity among all prepared electrodes. Hence, we proceeded to evaluate this condition for glucose‐sensitive film preparation.

The selected biosensor interface underwent evaluation for operational amperometric sensitivity and response stability. Five electrodes were produced for this purpose, undergoing a series of amperometric analyses to calculate sensitivity values, each lasting 1200 s. The sensitivity value of every electrode was determined by adding 10 µM glucose to the reaction medium five times, incrementally increasing the glucose concentration from 0 to 50 µM in the reaction cell. This process was repeated 12 times, resulting in 60 glucose measurements for each electrode. In total, the electrodes were operated for 4 h. Sensitivity values, initially set at 100% (Figure 3e2), were monitored to assess the deviation through repeated use. Results showed a maximum of 15% decrease in sensitivity values, indicative of good precision and stability. The average relative sensitivity (%) and relative standard deviation (RSD) (%) for each electrode were as follows: E1–97.97 and 6.21, E2–95.79 and 4.31, E3–92.08 and 5.19, E4–102.41 and 2.35, E5–101.85 and 3.96.

We then tested the electrode in a synthetic urine‐spiked medium (Figure 3e3). After adding 7.5 µM glucose to the reaction cell containing 9.5 mL PBS and waiting for the baseline to stabilize, the reaction volume was increased to 10 mL by adding 0.5 mL of synthetic urine, yielding 1:20 dilution. This dilution ratio keeps the pH value constant at 6 (< ±0.05). The calculated recoveries of 95.89% ± 3.16% (RSD = 3.29%) and 96.92% ± 2.80% (RSD = 2.89%) for the first and second additions of the same glucose level showed no significant interference from synthetic urine. Even with increasing glucose concentrations (30, 45, and 75 µM), the electrodes maintained high recoveries (94.25%–96.27%) and low RSDs (1.78%–4.68%) (Table S1, Supporting Information).

Focusing on achieving a sustainable and durable sensor interface, we also determined the hetero‐interface's shelf life by evaluating its amperometric signals with glucose additions over 7 days. On the 7th day, the electrodes maintained 96% of their initial electroactivity, demonstrating exceptional durability and stability throughout the testing period (Figure S6, Supporting Information).

In light of these examinations, which confirm its suitability for sensor production, the next section dissects the applicability of the optimized PB‐CHIA for LIG‐based POC prototype fabrication.

### 2.2 Fluidic Cell Module Design for the Prototype

PB‐CHIA adheres firmly to the GC surface despite its limited 2D contact area, thanks to its synthesis method. When moving from the GC substrate to a nano‐textured LIG electrode surface, PB‐CHIA interfaces in 3D, adding extra superficial porosity on a macroscopic scale to its original nanoscale porosity. The sensor responses can be modulated by the yielding interface between PB‐CHIA and the LIG surface, which can be varied according to the different surface morphologies produced by laser engraving at different power levels.

On the other hand, fully laser‐engraving the electrode surfaces and connection lines can streamline mass production and reduce the costs of fabricating LIG‐based POC devices.^[^ ^67^ ^]^ However, the engraved length of the working electrode's connection lines can introduce extra resistance, which in turn affects the sensor response.

**Figure 4:**
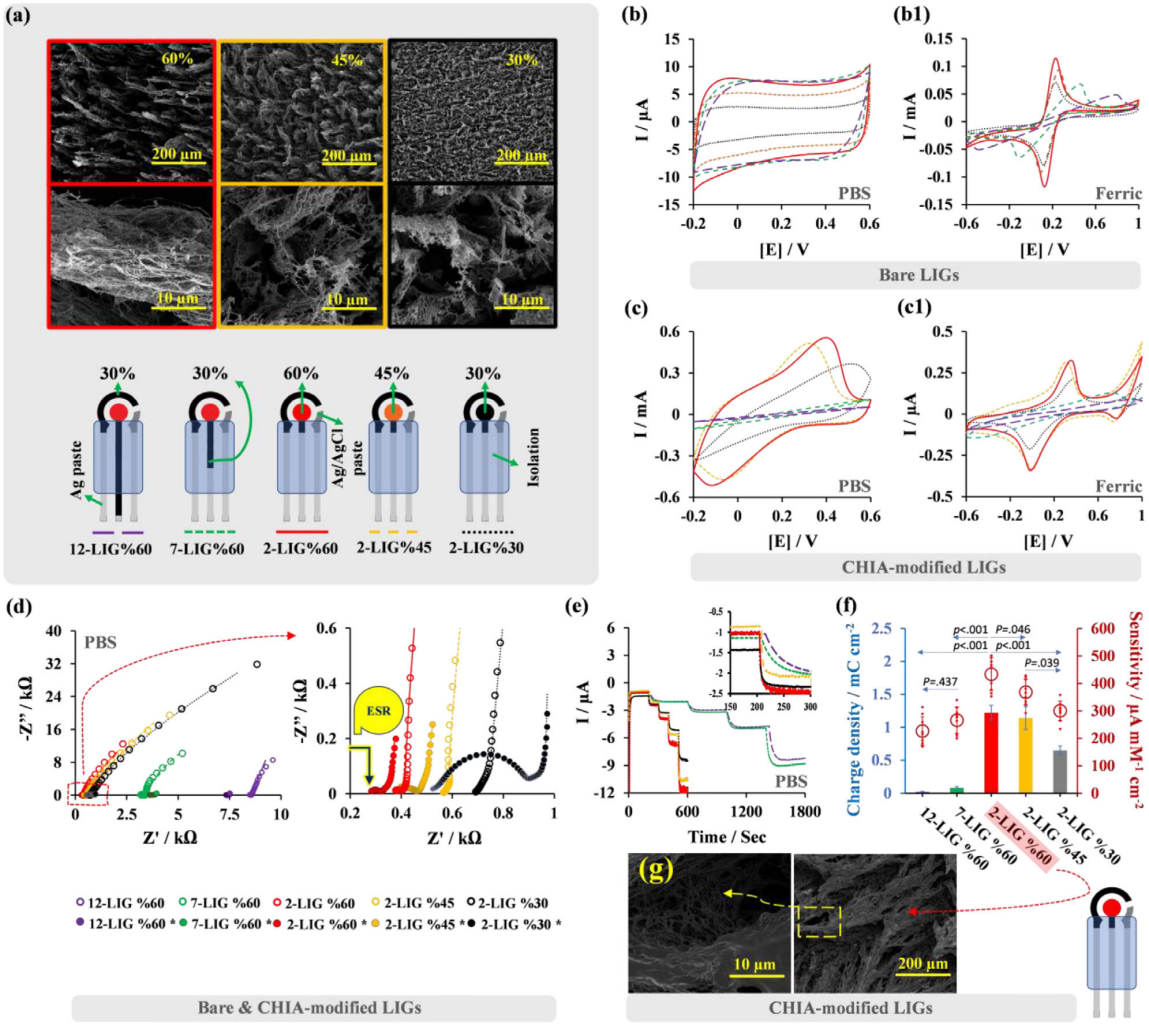
a) Various bare LIG surface designs on polyimide using 60% (red‐colored area), 45% (orange‐colored area), and 30% laser power (black‐colored area). The black‐colored tracks prepared with 30% laser power depict safety margins of 12, 7, and 2 mm in length. Reference electrodes are printed with Ag/AgCl paste. The counter electrode is prepared with 30% laser power. Starting from the connection point of the working electrode, the circuitry region is covered with a dielectric layer. Circuitry tracks are printed using Ag paste. b) their CV‐redox signals in PBS (scan rate: 0.05 V s^−1^) and b1) in ferric solution (scan rate: 0.1 V s^−1^), c) PB‐CHIA‐modified LIGs’ CV‐redox signals in PBS (scan rate: 0.05 V s^−1^) and c1 in ferric solution (scan rate: 0.1 V s^−1^). d) EIS assays of bare and PB‐CHIA‐modified LIGs (LIG*) in PBS (between 1×10^3^ and 0.1 Hz, E_dc_ = 0.0 V E_ac_ = 0.2 V, equilibration time = 5 s). e) Amperometric response of PB‐CHIA‐modified LIGs to H_2_O_2_ (Applied potential: −0.05 V) and f) corresponding charge density and sensitivity values. g) Surface morphology of 2‐LIG_%60_*. (For EIS assays, *n* = 3. In the figures, column plots represent average charge density values (*n* = 3), while hollow circles indicate average sensitivity values (*n* = 9). Error bars show the standard deviation of these averages. Data points depict individual sensitivity measurements within each dataset. *p*‐values are calculated using one‐way ANOVA followed by post‐hoc Tukey's HSD tests).

To investigate and optimize these parameters before designing the fluidic cell module, we varied the working electrode surface laser‐engraving powers to 30%, 45%, and 60% and set safety margins of 2, 7, and 12 mm between the working electrode surface and the Ag tracks for the circuitry. To prevent potential noise and interference from Ag tracks directly connected to the working electrode, we ensured the safety margin did not go below 2 mm (**Figure** **4a**).

These variations impacted parameters such as ∆E_a‐c_ (E_a_‐E_c_), ∆I_a‐c_ (I_a_‐I_c_), equivalent series resistance (ESR, composed of both ohmic and internal resistance ^[^ ^68^, ^69^, ^70^ ^]^ among other aspects^[^ ^71^ ^]^) and charge density values on the resulting sensor platform. We analyzed these variations to identify the optimal LIG‐based working electrode configuration and its safety margin to maximize sensor performance. This optimization is crucial before proceeding with the design of the flow cell module, which will enable the development of a POC prototype after incorporating an NFC system for the SMUG application.

Section 1.4.3 of the Supporting Information file detailed how we replicated the classical three‐electrode system on polyimide. We printed the reference electrode and its safety margin using Ag/AgCl ink with a PCB printer. The counter electrode and its safety margin on the polyimide substrate were also created via laser engraving at a 30% power level. By connecting each electrode's safety margins with Ag tracks for the potentiostat connection, we ensured that the resistance affecting the sensor responses primarily stemmed from the working electrode and its safety margin.

Figure 4b,c,b1‐c1 depict the CV‐redox curves for bare and PB‐CHIA‐modified LIG platforms in PBS and ferric solutions. Figure 4d presents EIS plots for these electrodes in PBS. Detailed equivalent circuit models are shown in Figure S7 (Supporting Information) and discussed in Section 2.2.1 of the Supporting Information.

#### 2.2.1 Effect of Working Electrode's Safety Margin's Length

Keeping the laser power level used for working electrode surface patterning the same and decreasing the safety margin from 12 to 7 and 2 mm (12‐LIG_60%_, 7‐LIG_60%,_ and 2‐LIG_60%_) results in an increase in ∆I_a‐c_ values (from 0.099 to 0.131 and 0.232 mA) and decrease in ∆E_a‐c_ values (from 1.279 to 0.568 and 0.108 V) in ferric solution (for all cases CV‐redox currents and peak potentials in PBS and ferric solution, 5 mm of Fe[(CN)_6_]^3‐/4−^ + 0.1 M KCl, refer to Tables S2–S4, Supporting Information). This trend is supported by decreased ESR (R_1_) from 8.331 to 3.237 and 0.324 kΩ (for all cases’ EIS findings, refer to Tables S5 and S6, Supporting Information). Therefore, as the working electrode safety margin shortens, these bare electrodes in PBS show more pronounced capacitive CV‐redox curves, and the charge density value increases from 10.030 to 10.266 and 13.618 µC cm^−2^.

When (PEDOT:PB)G modifies the 12‐LIG_60%_, 7‐LIG_60%,_ and 2‐LIG_60%_ platforms, ∆I_PB‐PW_ value increases from 0.135 to 0.270 and 0.695 µA and the ∆E_PB‐PW_ value decreases from 1.355 to 1.212 and 0.334 V in ferric solution. This trend is accompanied by decreased ESR values from 7.052 to 3.434 and 0.266 kΩ. The high ESRs cause the E_PB_ and E_PW_ peaks to fall outside the working potential window for the (PEDOT:PB)G‐modified 12‐LIG_60%_ and 7‐LIG_60%_ in PBS. However, with a 2 mm safety margin, clear E_PB_ and E_PW_ peaks are observed in PBS, yielding ∆I_PB‐PW_ (1.078 mA) and ∆E_PB‐PW_ (0.516 V). The charge density value with the 2 mm safety margin for 2‐LIG_60%_ in the PBS solution is determined as 13.618 µC cm^−2^.

#### 2.2.2 Effect of Laser Power Level for Working Electrode Surface Engraving

For the case of maintaining a consistent 2 mm safety margin for all platforms and reducing the power level of the working electrode surface from 60% to 45%, 30% (2‐LIG_60%_, 2‐LIG_45%_, and 2‐LIG_30%_) results in a decrease in ∆I_a‐c_ values (from 0.232 to 0.185 and 0.149 mA) with slight ∆E_a‐c_ value oscillations (0.108, 0.174, and 0.119 V) in ferric solution. In parallel, the calculated ESR values increase (from 0.324 to 0.556 and 0.691 kΩ) while the charge densities diminish (from 13.618 to 7.080 and 2.93 µC cm^−2^).

When 2‐LIG_60%_, 2‐LIG_45%_, and 2‐LIG_30%_ platforms are modified with (PEDOT:PB)G, those platforms displayed again decreasing ∆I_PB‐PW_ values (0.695, 0.652, and 0.418 mA) and oscillating but clear E_PB_ and E_PW_ peaks (yielding ∆E_PB‐PW_ values of 0.334, 0.305 and 0.389 V) in ferric solution. Similarly, while the ∆I_PB‐PW_ values (1.078, 0.981, and 0.678 mA) decrease, the ∆E_PB‐PW_ value (0.516, 0.369, and 0.673 V) shows an oscillating trend in PBS. In parallel, ESR values (0.266, 0.343, and 0.504 kΩ) varied inversely with the charge density values (1.222, 1.142, and 0.652 mC cm^−2^).

#### 2.2.3 Nano‐textured Working Electrode Design: LIG as Nano‐fibrous Fringes for Fluidic Cell Module

Figure 4e illustrates amperometric responses to H_2_O_2_ across (PEDOT:PB)G‐modified LIG‐based platforms, while Figure 4f compiles their sensitivity and calculated charge density values according to the varied factors, such as the engraving power level of the working electrode and its safety margin length. The reduction in the safety margin has an effect on the system resistance that leads to decreased ESR and ∆E_PB‐PW_, as well as increased ∆I_PB‐PW_ and charge density values. Conversely, lowering the power level for engraving the working electrode increases ESR and decreases ∆I_PB‐PW_ and charge density values, with ∆E_PB‐PW_ values showing oscillations. Although the 2‐LIG_60%_ platform appears to be the optimal sensor platform to use considering ESR, ∆I_PB‐PW_, and charge density values influenced by safety margin and power level variations, it is necessary to crosscheck the amperometric responses to fully evaluate its compatibility with (PEDOT:PB)G and determine the most effective sensor platform.

As seen in Figure 4e, the LIG platforms with the shortest safety margin always generate responses faster and higher than others. Our findings imply that the 60% engraving power level is the best for working electrodes with the shortest safety margin (2‐LIG_60%_) for modification with (PEDOT:PB)G, resulting in an H_2_O_2_ sensitivity of 433.330 µA mM⁻¹ cm⁻^2^. This sensitivity significantly outperforms those obtained from 12‐LIG_60%_ and 7‐LIG_60%_ (226.34 and 265.68 µA mM⁻¹ cm⁻^2^, respectively) and 2‐LIG_45%_ and 2‐LIG_30%_ (368.15 and 300.15 µA mM⁻¹ cm⁻^2^, respectively), as confirmed by post‐hoc Tukey's HSD analyses with *p*‐values less than 0.001 in most comparisons (Figure 4f). Using 2‐LIG_60%_ (hereafter referred to as LIG‐F) instead of GC as a working electrode substrate has significantly enhanced the linear range and sensitivity of the PB‐CHIA's electroanalytical performance, thanks to nano‐fibrous fringes acting as nanoelectrodes, which enhance charge transfer between the solid and liquid junction due to increased film porosity and the contact surface between the polymer composite and the working electrode surface (Figure 4g). A complementary electrochemical comparison in terms of CV‐redox and ESI characteristics (Figure S8 and Tables S7–S10, Supporting Information) between GC and LIG‐F electrodes is discussed in SI's Section 2.2.2.

**Figure 5:**
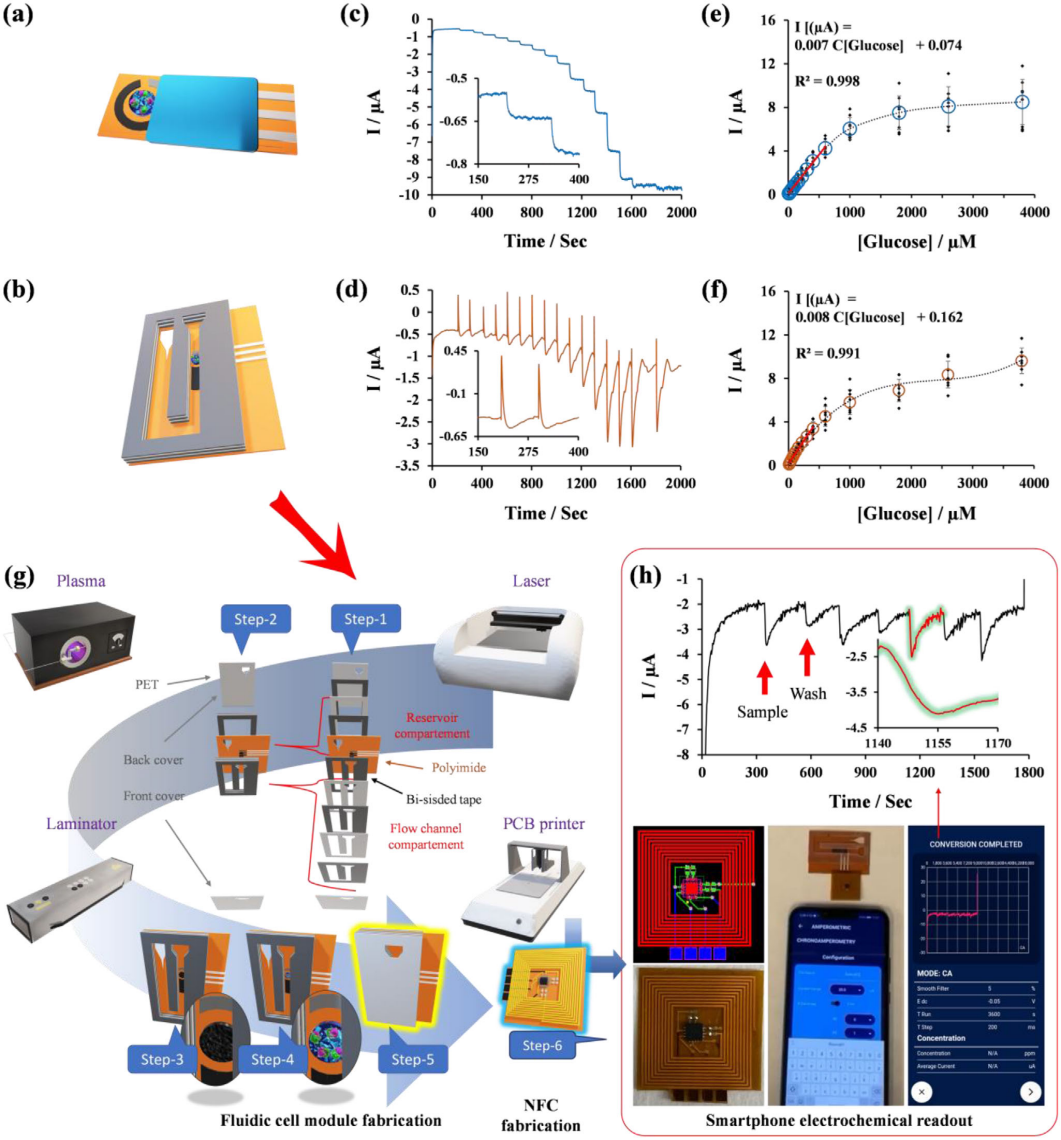
Quantification of LIG‐F‐based three‐electrode platform and fluidic cell: Prototype fabrication and validation. a, b) LIG‐F‐based platform [LIG‐F/(PEDOT:PB)G‐GOx] and fluidic cell [LIG‐F_FC_/(PEDOT:PB)G‐GOx], c, d) Amperometric responses to successive glucose injections (Inset: magnified view of the first two injections, 10 and 20 µM), and e, f) corresponding calibration plots between 10 and 4000 µM glucose. g) Fabrication of fluidic cell and h) assembly of the NFC potentiostat read mode with a smartphone and its real‐time glucose readout (inset curve showing amperometric signals of the four consecutive additions of the same sample containing glucose and intermittent rinsing with PBS) in synthetic urine‐spiked samples (supplementary video1). (Applied potential is −0.05 V for amperometric measurements in PBS. In e and f plots, circles represent each addition's average response values (*n* = 9), with error bars indicating the standard deviation. The linear response regions of the sensor to analyte additions (where R^2^ > 0.990) are marked by a solid red line).

After identifying the best condition for the LIG (LIG‐F), we modified it with (PEDOT:PB)G‐GOx, similar to what was done with GC electrodes. Glucose detection as a standalone platform using LIG‐F/(PEDOT:PB)G‐GOx under stirring conditions (immersed in a reaction cell) (**Figure** **5**a) showed a sensitivity of 25.39±2.98 µA mM^−1^ cm^−2^ with an limit‐of‐detection (LOD) of 1.53±1.40 µm within a wider linear range of 10–600 µm (“I(µA) = 0.007C_[Glucose]_+0.074”, R^2^: 0.998) (Figure 5c,e), outperforming the GC electrode‐based biosensor (Figure 3d1–d2) (48.62±3.42 µA mM^−1^ cm^−2^ within 10–160 µm, with LOD of 1.16±0.47 µm glucose) in terms of the linear range. It is speculated that the engraved electrode surface, featuring nano‐fringe multi‐electrodes, increased the total active area and facilitated electron transfer paths. These enhancements, combined with the boosting properties of graphene‐based materials,^[^ ^72^ ^]^ likely contributed to the extended working range for glucose detection. Besides, the relatively lower sensitivity value compared to GC‐based biosensors may be attributed to the side effects of the enzyme immobilization process, which might decrease the superficial porosity of the (PEDOT:PB)G‐modified LIG‐F (Figure S9, Supporting Information). The nanocomposites deposit loosely onto the large surface area of the LIG‐F (Figure 4g), compared to the dense structures from on GC (Figure 2b). After the addition, the glutaraldehyde can penetrate more effectively into the porous surface, leading to a more efficient cross‐linking between polymer, gelatine, and enzyme at the molecular level, having an easier pathway to reach even the deeper levels of the matrix.

#### 2.2.4 Fluidic Cell Module Quantification, Selectivity, Interference, and Validation in Synthetic Urine Samples

##### 2.2.4.1 Quantification

The LIG‐F‐based biosensor was then integrated into a fluidic cell module (LIG‐FFC, Figure 5b) using a combination of simple lab and office equipment (Figure 5g) and calibrated with glucose additions (Figure 5d,f). When a sample is added to the inlet of the fluidic cell module, it moves through the fluidic cell channel by surface hydrophilic attraction, reaching the three‐electrode compartment. Each subsequent sample added for further analysis pushes the previous sample forward with the help of hydrophilic attraction, also reaching the three‐electrode compartment. This process allows for the sequential addition and analysis of multiple samples. Operated via limitless batch‐flow‐injection mode (gravity‐assisted), injecting the samples into the inlet of the module (a napkin behind its reservoir tilted at a 75° angle), the LIG‐F_FC_ performed in a wider linear range, 10–400 µm (“I(µA) = 0.008C_[Glucose]_ + 0.162”, R^2^: 0.991) compared to the GC electrode‐based biosensor. The sensitivity was 29.88 ± 4.98 µA mM^−1^ cm^−2^, with 4.52 ± 2.24 µm LOD.

##### 2.2.4.2 Selectivity, Interference, and Operational Stability of the Fluidic Cell

The selectivity and interference tolerance of the sensor were evaluated using a model urine sample, and its performance was further validated in synthetic urine. Limited batch‐flow‐injection mode was used, as detailed in Figure S11 and Section 2.3 of Supporting Information.

When the model urine sample was diluted to 1:20, it contained 7.5 µM ascorbic acid, 2.5 µM uric acid, 500 µM urea, 500 µM NH_4_^+^, and 50 µM creatinine.^[^ ^73^, ^74^ ^]^ The individual contributions of these interferants to the sensor response, normalized to the response for 100 µM glucose (100%), were 9.81%, 14.02%, 14.08%, 18.33%, and 10.88%, respectively. For 25 µM glucose alone, the sensor produced a signal corresponding to 26.33% of the response for 100 µM glucose. In the presence of all the above interferants, the total response for 25 µM glucose (0.45 mg dL^−1^) increased to 30.84%, representing a 17.12% increase (0.077 mg dL^−1^) due to the interferants.

Although no standardized error threshold exists for urine glucose measurements, the observed deviation falls within the ±20% range commonly considered clinically acceptable in blood glucose monitoring. According to Clarke Error Grid Analysis, deviations within this range are classified in Zones A or B, indicating minimal clinical risk ^[^ ^75^, ^76^ ^]^. Additionally, ISO 15 197:2013 guidelines define an accuracy threshold of ±15 mg dL^−1^ for glucose concentrations <100 mg dL^−1^, which our sensor's observed deviation remains well below.^[^ ^77^ ^]^

To further validate the sensor, glucose detection was performed in synthetic urine. Within the 30–100 µM glucose working range, the sensor achieved recovery values of 90.81%–106.62% and RSD values of 1.12%–4.25% when used with 1:20 diluted synthetic urine in PBS (Table S11, Supporting Information).

The operational stability of the fluidic cell‐based sensors was assessed over a 7‐day period. The sensitivity values of three independently fabricated sensors, which were concurrently employed in additional analyses throughout the period, were recorded on the first and seventh days under identical measurement conditions. The average sensitivity decreased from 28.42 ± 5.48 to 26.13 ± 3.28 µA mM^−1^ cm^−2^, corresponding to 92% retention over the 7‐day period. This indicates that the sensors maintain an acceptable level of stability under practical usage scenarios, with sensitivity values remaining close to those obtained during calibration (29.88 ± 4.98 mM^−1^ cm^−2^).

These results indicate that the fluidic cell‐based sensors exhibit strong selectivity and minimal interference effects, supporting their applicability for non‐invasive glucose monitoring in complex biological matrices, with a stability level suitable for practical applications.

### 2.3 Prototype: Fully Integrated Battery‐Free & Wireless Electrochemical Readout

After fabricating the fluidic cell module using laboratory equipment, a flexible printed circuit board (PCB) consisting of an antenna and a single‐chip potentiostat, SIC4341, is attached (Figure 5g,h). We validated the constructed prototype following the operational guide in SI (Section 2.3). The integration of the NFC system enabled us to monitor glucose through a smartphone. The single‐chip potentiostat, with NFC technology, enables wireless power and data transmission, facilitating the use of various electrochemical techniques, including amperometry (Figure S12, Supporting Information). This makes it an excellent alternative for wearables and portable devices.^[^ ^78^ ^]^ Here, we will only discuss the results of the amperometric analysis, which can be implemented in a real‐life scenario. Further details regarding the antenna's design and specifications can be found in the Supporting Information (Section 2.4).

The fluidic cell module has a capacity of 400 µL, including the flow channel, and 50 µL samples for each reading, which is enough to run the prototype, regardless of volume variations, as long as the sample reaches the three‐electrode compartment. The flow channel and the reservoir can be washed with the recommended buffer solution and reused several times for up to a week.

As discussed before, the fluidic cell module of the prototype linearly responds to glucose concentrations within the 10–400 µM linear range, with a calculated LOD of ≈4.5 µM. When adjusting these values for the dilution factor, it works within 300–8×10^3^ µM glucose, which covers the medical normal 0–0.25 mg dL^−1^ (equivalent to 0–1.4 × 10^3^ µM) ^[^ ^79^ ^]^ and higher glucose concentration in urine up to 8 × 10^3^ µM. The module's LOD value is as low as 90 µM. The difference between the blank and amperometric peak reduction currents can be converted to µM glucose in the sample by using the re‐arranged equation of C_[Glucose]_ = {[I(µA)‐0.162]x20}/0.008, (R^2^: 0.991).

Typically, a one‐time sample addition is enough to measure the glucose content of the added sample. However, to show the prototype's ability (Supplementary video1) to run the measurements continuously, employing the batch‐injection mode, the 1:20 diluted synthetic urine sample containing 200 µM glucose was added four times from the inlet of the fluidic cell, and the flow channel was rinsed intermittently with blank PBS. The prototype operates with high‐speed amperometric readings in 15 s and accurately detected 200 µM glucose with an impressive recovery (99.99%) and low RSD (4.9%) values (*n* = 4). It is worth noting that the observation of signals during the washing process is caused by residual glucose from previous samples existing between the sample inlet and the working electrode. While the washing solution displaces this residue, the working electrode surface encounters remnants of the previous glucose‐containing sample that generate a reduction current. However, once the displacement stops, the trace amount of glucose, if it still exists on the working electrode, is consumed, and the reduction currents turn back to the former baseline, indicating system readiness for the next sample addition.

**Table** **1** compares our prototype with other electrochemical sensor electrodes and POCs for urine glucose monitoring, considering wearability, portability, and nanomaterial composition. Our prototype, fabricated with standard laboratory equipment and techniques, shows comparable electroanalytical performances and stands out as the sole fully integrated, battery‐free, wireless smartphone electrochemical readout prototype for SMUG in the literature.

**Table 1:** Comparison of the current study vs selected amperometric sensors from the literature.

Electrode	Measurement Medium Counter Electrode Applied Potential	Linear range µM^−1^	Sensitivity µA mM^−1^ cm^−2^	LOD µM	Long‐Term stability	Potentiostat Power source Display	Physiological Sample POC Type	Refs.
SPCPE/Ru‐NCst/GOx	NA	NA	NA	NA	No significant change in sensor signal by day 66	‐Custom‐designed ‐Needs battery ‐Custom‐designed	‐Urine (Type is NA) ‐Self‐monitoring portable	[13]
PCB‐Au/FGO/Silane/GOx‐Nf‐Silane	Human Urine and TES buffer, pH: NA Ct: Au +0.6 V^d)^	0‐24 × 10^3^	NA	NA	Sensor signal decreased to 8.7% of the initial value after 30 days	‐Custom‐designed ‐NA ‐NA	‐Human urine ‐Self‐monitoring portable or stationary	[12]
SPCE/rGO/CuO‐IL/NCWPP	0.1 m PBS, pH: 6 Ct: C‐ink/paste +0.4 V^b)^	0.03‐7 × 10^3^	21.26	0.19	Sensor retained 92% of the initial signal after 30 days of storage	‐Commercial ‐No need ‐Smartphone	‐Human urine ‐Self‐monitoring portable	[14]
Anode^+/−^: PET/Ag‐ink/SPCE/MWCNT‐AuNPs‐GOx	0.1 m PBS, pH: 5.3‐8 Ct: Pt‐wire 1.8 V^a)^	0.2 × 10^3^‐5 ×10^3^	NA	NA	Output power remained at 90% of the original value after 5 days	‐Enzymatic fuel cell ‐No battery needs ‐LED light intensity	‐Synthetic Urine ‐Wearable	[9]
PET/Ag‐ink/SPCE/TTF/GOx	0.1 m PBS, pH: 7.4 Ct: C‐ink/paste +0.1 V^b)^	0‐2 × 10^3^ 2 × 10^3^‐20 ×10^3^	22.1 × 10^−3^ µA mM^−1^ 63.5 × 10^−3^ µA mM^−1^	NA NA	Sensor response current maintained at 91.1% of initial value after 5 days	‐Commercial ‐Needs a power source ‐Smartphone	‐Human Urine ‐Wearable	[10]
PET/Au‐sputter/CNT/Pt/GOx/Nf	(NA) m PBS, pH: 7.2 Ct: Pt‐sputter +0.4 V^b)^	1 × 10^3^‐14 × 10^3^	2.71 µA mM^−1^	15.5	No significant change in sensor signal by day 6	‐Custom‐designed ‐Needs battery ‐Smartphone	‐Human Urine ‐Wearable	[11]
SRcP/s‐Au/AuNPs/PB/GOx/Nf	0.01 M PBS, pH 7.4 Ct: s‐Au −0.1 V^b)^	50‐1000	21.3	30	The glucose sensor maintained 82.68% of initial response after 4 weeks	‐NA ‐NA ‐Computer screen	Artificial urine, human urine ‐self‐monitoring	[80]
GC/PBPPyBA	0.05 m PBS + 0.1 m KCl pH: 6 Ct: Pt‐wire 0.0 V^c)^	100–1000	2.5	10	Initial sensitivity decreases within the first 3–4 measurements, with <5% variation over 10 days	‐Commercial ‐Needs a power source ‐Computer screen	‐Human Urine, several other samples ‐Working electrode	[81]
GC/Au‐PAA/PB/GOx	0.2 m PBS, pH 6.9 Ct: Pt‐wire −0.1 V^c)^	5‐8 × 10^3^	445 uA m ^−1^ or (3.5* based on GC surface area)	1	Sensor signal decreased by 15% after 4 weeks	‐Commercial ‐Needs a power source ‐Computer screen	‐Human Blood and Urine ‐Working electrode	[30]
PLA‐PEG/SPCE/PB/GOx	Synthetic Urine + 0.1 M KCl, pH: 6.0 Ct: C‐ink/paste 0 V^b)^	500‐5240	24	197	No significant change in sensor signal by day 60 (SD 12%)	‐Commercial ‐Needs a power source ‐Computer screen	‐Synthetic Urine −3E‐System	[82]
GC/(PEDOT:PB)G‐GOx	0.05 m PBS + 0.1 m KCl pH: 6 Ct: Pt‐wire −0.05 V^a)^	10‐160	48.62 ± 3.42	1.16 ± 0.47	Preserved 96% of the initial current signal by day 7	‐Commercial ‐Needs a power source ‐Computer screen	‐Synthetic Urine ‐Working electrode	This study
LIG‐F/(PEDOT:PB)G‐GOx	0.05 m PBS + 0.1 m KCl pH: 6 Ct: LIG −0.05 V^b)^	10‐600	25.39 ± 2.98	1.53 ± 1.40	Not studied	‐Commercial ‐Needs a power source ‐Computer screen	‐PBS −3E‐System in fluidic cell module	This study
LIG‐F_FC_/(PEDOT:PB)G‐GOx	0.05 m PBS + 0.1 m KCl pH: 6 Ct: LIG −0.05 V^b)^	10‐400	29.88 ± 4.98	4.52 ± 2.24	Preserved 92% of the initial current signal by day 7	‐Custom‐designed ‐No need ‐Smartphone	‐Synthetic Urine ‐Self‐monitoring portable	This study

^+/−^ Cathode: PET/SPCE/ MWCNT‐MnO_2_, AuNPs: Gold nanoparticles, 3E‐System: Three‐electrode system, 3‐APTES: 3‐aminopropyltriethoxysilane, C‐ink/paste: Carbon ink or paste, Ct: Counter electrode, G: Gelatin, GA: Glutaraldehyde, GC: Glassy carbon electrode, GO: Graphene oxide, GOx: Glucose oxidase, Gp: Graphite electrode, Gr: Graphene, GS: Graphene sponge, IL: Ionic liquid (1‐butyl‐2,3‐dimethylimidazolium tetrafluoroborate), LIG: Laser‐induced graphene, LIG‐F: Laser‐induced graphene as nano‐fibrous fringes, LIG‐F_FC_: LIG‐F‐based fluidic cell, MPHs: Metalloid polymer‐complex (Polyethylene glycole and Silver‐Silica material), NA: Not available, NCWPP: NaOH‐coated wax‐printed paper, Nf: Nafion, PAA: porous anodic alumina, PB: Prussian blue nanoparticles, PBS: Phosphate buffered saline, PEDOT: Poly (3,4‐ethylene dioxythiophene), PET: Polyethylene terephthalate, PI: Polyimide, PPyBA: 4(pyrrole‐1‐yl) benzoic acid, Pt: Platin electrode, rGO: Reduced graphene oxide, Ru‐NCst: ruthenium (III) chloride‐nitrocellulose strip, s‐Au: Gold‐sputtered electrode, SPCPE: Screen‐printed carbon paste, SRcP: Silicone rubber coated paper, TTF: Tetrathiafulvale;

^a)^ vs Ag/AgCl reference (KCl_aq_);

^b)^ vs Ag/AgCl ink, paste, or others‐based reference;

^c)^ vs Saturated calomel reference;

^d)^ vs Au‐based reference.

## 3 Conclusion

In this study, we explored a polymer‐based strategy to improve the operational stability of PBNPs, the most effective H_2_O_2_‐selective nanocatalyst for biosensor development. Our single‐step approach, which supports the mass production of working electrodes, offers simplicity and robustness for sensor production compared to complex or multi‐stage techniques found in the literature. The operational stability of PBNPs was enhanced by incorporating their precursors into a conductive polymer (PEDOT) and the functional groups of a soft template (gelatin) during chemical polymerization. This process yielded a self‐assembled PB‐CHIA, where PBNPs were covered with a physical blockage layer composed of PEDOT and gelatin. Further enhancement was acquired by cross‐linking glucose GOx onto the composite using glutaraldehyde, which forms hydrophobic pockets around the PBNPs. The resulting composite was then evaluated for SMUG applications for diabetes management.

We tested the electroanalytical performance of PB‐CHIA on both 2D GC electrode substrates and 3D LIG surfaces obtained by laser ablation of the polyimide substrate. The performance of the resulting glucose biosensors was compared after simply immobilizing the GOx enzyme on the PB‐CHIA‐modified working electrodes via glutaraldehyde. Additionally, in designing the fluid cell module on a polyimide substrate, we found that the LIG‐based safety margin of the electrical lines connecting the working electrode to the potentiostat impacts sensor performance and must be considered for the sensor platforms reliant on laser ablation of polymeric substrates.

The optimal LIG morphology, nano‐fibrous fringes (LIG‐F, diameter: 72.87 ± 12.24 nm), which maximized the electroanalytical performance of PB‐CHIA and surpassed the GC‐based sensors, was used for the design of the fluidic cell module, allowing for the addition of multiple samples. With this configuration, the biosensor exhibited a linear range of 10–400 µM (R^2^: 0.991, sensitivity: 29.88 ± 4.98 µA mM⁻¹ cm⁻^2^, LOD: 4.52 ± 2.24 µM) after GOx immobilization. It accurately detected glucose in 1:20 diluted synthetic urine. Within the studied range of 30–100 µM, it performed recovery rates of 90.81%–106.62% and low RSDs of 1.12%–4.15%, ensuring reliability for both normal and elevated urine glucose levels, making it a potential home‐based SMUG option.

The fluidic cell module was combined with a custom‐designed NFC system, which made it possible to detect glucose in the synthetic urine within 15 s via a smartphone. The device achieved ≈99% recovery value with a low RSD of %4.9 (*n* = 4) when detecting glucose in synthetic urine samples diluted 1:20 with PBS. The batteryless, wireless operation facilitated by the NFC module ensures safe and comfortable usage by preventing contact between the phone and the sensor used with urine samples, thus avoiding potential sample leakage onto the phone and its electrical circuits.

With its extended operational stability, the suggested material, PB‐CHIA's sensitivity and selectivity to H_2_O_2_, and its enzyme cross‐linking sites present potential for developing diverse POC devices for urinary analysis by accommodating other oxidase‐based enzymes. Aiming to replace single‐use sensors, our study introduces the first battery‐free, wireless prototype for SMUG through smartphones, representing a significant leap toward making portable healthcare more accessible pending successful clinical validation with real samples.

## 4 Experimental Section

Readers should refer to the Supporting Information for details on chemicals, PB‐CHIA synthesis, prototype specifications, electrode modifications, supplementary explanations, tables, and figures that complement the main text.

## Conflict of Interest

The authors declare no conflict of interest.

## Author Contributions

S.U. performed conceptualization, investigation, validation, methodology, formal analysis, visualization, writing, original draft, writing, review and editing, and project administration. G.M. performed investigation, validation, methodology, formal analysis, writing, review, and editing. M.U. performed investigation, validation, methodology, formal analysis, writing, review, and editing. A.M. performed conceptualization, investigation, validation, methodology, formal analysis, writing, review, and editing, funding acquisition, resources, project administration, supervision

## Supporting information



Supporting Information

Supplementary Video1

## Data Availability

The data that support the findings of this study are available from the corresponding author upon reasonable request.

## References

[1] Y. Liu , Q. Yu , X. Luo , L. Yang , Y. Cui , Microsyst. Nanoeng. 2021, 7, 75. 34631143 10.1038/s41378-021-00302-w PMC8481261

[2] Y. Zou , Z. Chu , J. Guo , S. Liu , X. Ma , J. Guo , Biosens. Bioelectron. 2023, 225, 115103. 36724658 10.1016/j.bios.2023.115103

[3] D. A. Lawlor , A. Fraser , R. S. Lindsay , A. Ness , D. Dabelea , P. Catalano , G. D. Smith , N. Sattar , S. M. Nelson , Diabetologia 2010, 53, 89. 19841891 10.1007/s00125-009-1560-z

[4] S.‐S. Yang , P. Chu , Y.‐F. Lin , A. Chen , S.‐H. Lin , Am. J. Kidney Dis. 2002, 39, e14.1. 11877594 10.1053/ajkd.2002.31425

[5] J. Calado , R. Santer , J. Rueff , Kidney Int. 2011, 79, S7. 10.1038/ki.2010.510 21358700

[6] V. K. Mahajan , N. L. Sharma , R. C. Sharma , G. Garg , Int. J. Dermatol. 2005, 44, 821. 16207182 10.1111/j.1365-4632.2005.02218.x

[7] C.‐C. Hung , H. Y.‐H. Lin , J.‐J. Lee , L. M. Lim , Y.‐W. Chiu , H.‐P. Chiang , S.‐J. Hwang , H.‐C. Chen , Sci. Rep. 2016, 6, 39372. 28008953 10.1038/srep39372 PMC5180243

[8] T.‐T. Wang , X.‐F. Huang , H. Huang , P. Luo , L.‐S. Qing , Adv.Sens. Energy Mater. 2022, 1, 100016.

[9] J. Zhang , J. Liu , H. Su , F. Sun , Z. Lu , A. Su , Sens Actuators B Chem 2021, 341, 130046.

[10] H. Su , F. Sun , Z. Lu , J. Zhang , W. Zhang , J. Liu , Sens. Actuators, B 2022, 357, 131459.

[11] X. Li , C. Zhan , Q. Huang , M. He , C. Yang , C. Yang , X. Huang , M. Chen , X. Xie , H.‐J. Chen , ACS Appl. Nano Mater. 2022, 5, 4767.

[12] H. Y. Kim , K. J. Jang , M. Veerapandian , H. C. Kim , Y. T. Seo , K. N. Lee , M.‐H. Lee , Biotechnol. Rep. 2014, 3, 49. 10.1016/j.btre.2014.06.003 PMC5466094 28626648

[13] H. D. Park , K. J. Lee , H. R. Yoon , H. H. Nam , Comput. Biol. Med. 2005, 35, 275. 15749089 10.1016/j.compbiomed.2004.02.003

[14] N. Janmee , P. Preechakasedkit , N. Rodthongkum , O. Chailapakul , P. Potiyaraj , N. Ruecha , Anal. Methods 2021, 13, 2796. 34114570 10.1039/d1ay00676b

[15] A. A. Karyakin , Electroanalysis 2001, 13, 813.

[16] R. Zhang , K. Fan , X. Yan , Sci. China Life Sci. 2020, 63, 1183. 31974863 10.1007/s11427-019-1570-7

[17] J. F. KEGGIN , F. D. MILES , Nature 1936, 137, 577.

[18] F. Ricci , G. Palleschi , Biosens. Bioelectron. 2005, 21, 389. 16076428 10.1016/j.bios.2004.12.001

[19] L. Gao , J. Zhuang , L. Nie , J. Zhang , Y. Zhang , N. Gu , T. Wang , J. Feng , D. Yang , S. Perrett , X. Yan , Nat. Nanotechnol. 2007, 2, 577. 18654371 10.1038/nnano.2007.260

[20] A. A. Karyakin , Curr. Opin. Electrochem. 2017, 5, 92.

[21] X.‐H. Liu , J. Peng , W.‐H. Lai , Y. Gao , H. Zhang , L. Li , Y. Qiao , S.‐L. Chou , Adv. Funct. Mater. 2022, 32, 2108616.

[22] J. Li , J.‐D. Qiu , J.‐J. Xu , H.‐Y. Chen , X.‐H. Xia , Adv. Funct. Mater. 2007, 17, 1574.

[23] Y. Xie , R.‐B. Lin , B. Chen , Adv. Sci. 2022, 9, 2104234. 10.1002/advs.202104234 PMC8728855 34825524

[24] R. Barber , J. Davis , P. Papakonstantinou , ACS Appl. Nano Mater. 2023, 6, 10290.

[25] A. A. Karyakin , E. E. Karyakina , L. Gorton , Electrochem. Commun. 1999, 1, 78.

[26] I. L. de Mattos , L. Gorton , T. Ruzgas , A. A. Karyakin , Anal. Sci. 2000, 16, 795.

[27] S. A. Jaffari , A. P. F. Turner , Biosens. Bioelectron. 1997, 12, 1.

[28] F. Ricci , A. Amine , G. Palleschi , D. Moscone , Biosens. Bioelectron. 2002, 18, 165. 10.1016/s0956-5663(02)00169-0 12485762

[29] F. Ricci , G. Palleschi , Y. Yigzaw , L. Gorton , T. Ruzgas , A. Karyakin , Electroanalysis 2003, 15, 175.

[30] Y. Xian , Y. Hu , F. Liu , Y. Xian , L. Feng , L. Jin , Biosens. Bioelectron. 2007, 22, 2827. 17188857 10.1016/j.bios.2006.11.020

[31] Z.‐D. Gao , Y. Qu , T. Li , N. K. Shrestha , Y.‐Y. Song , Sci. Rep. 2014, 4, 6891. 25367086 10.1038/srep06891 PMC4219169

[32] E. V. Karpova , E. E. Karyakina , A. A. Karyakin , RSC Adv. 2016, 6, 103328.

[33] E. V. Karpova , E. V. Shcherbacheva , M. A. Komkova , A. A. Eliseev , A. A. Karyakin , J. Phys. Chem. Lett. 2021, 12, 5547. 34101473 10.1021/acs.jpclett.1c01200

[34] P. Salazar , M. Martín , R. Roche , R. D. O'Neill , J. L. González‐Mora , Electrochim. Acta 2010, 55, 6476.

[35] L. V. Lukachova , E. A. Kotel'nikova , D. D'Ottavi , E. A. Shkerin , E. E. Karyakina , D. Moscone , G. Palleschi , A. Curulli , A. A. Karyakin , Bioelectrochemistry 2002, 55, 145. 11786361 10.1016/s1567-5394(01)00146-3

[36] P. Salazar , M. Martín , R. D. O'Neill , R. Roche , J. L. González‐Mora , J. Electroanal. Chem. 2012, 674, 48.

[37] N. K. , C. S. Rout , RSC Adv. 2021, 11, 5659. 35686160 10.1039/d0ra07800j PMC9133880

[38] L. Basabe‐Desmonts , D. N. Reinhoudt , M. Crego‐Calama , Chem. Soc. Rev. 2007, 36, 993. 17534482 10.1039/b609548h

[39] A. V. Borisova , E. E. Karyakina , S. Cosnier , A. A. Karyakin , Electroanalysis 2009, 21, 409.

[40] V. Seshadri , L. Wu , G. A. Sotzing , Langmuir 2003, 19, 9479.

[41] L. C. Chang , H. N. Wu , C. Y. Lin , Y. H. Lai , C. W. Hu , K. C. Ho , Nanoscale Res. Lett. 2012, 7, 1. 22716478 10.1186/1556-276X-7-319 PMC3475046

[42] M. Yang , Y. Liu , Y. Song , G. Zhao , H. Tan , Q. Zhang , F. Xu , Int. J. Electrochem. Sci. 2017, 12, 4428.

[43] S. Uzunçar , N. Özdoğan , M. Ak , Mater. Today Commun. 2021, 26.

[44] S. Uzunçar , N. Ozdogan , M. Ak , J. Electrochem. Soc. 2021, 168, 076509.

[45] S. Uzuncar , N. Ozdogan , M. Ak , Anal. Chim. Acta 2021, 1172, 338664. 34119021 10.1016/j.aca.2021.338664

[46] S. Uzunçar , H. Kaç , M. Ak , Talanta 2023, 252, 123841. 36001902 10.1016/j.talanta.2022.123841

[47] M. J. Donahue , A. Sanchez‐Sanchez , S. Inal , J. Qu , R. M. Owens , D. Mecerreyes , G. G. Malliaras , D. C. Martin , Mater. Sci. Engineer.: R: Rep. 2020, 140, 100546.

[48] A. Malti , J. Edberg , H. Granberg , Z. U. Khan , J. W. Andreasen , X. Liu , D. Zhao , H. Zhang , Y. Yao , J. W. Brill , I. Engquist , M. Fahlman , L. Wågberg , X. Crispin , M. Berggren , Adv. Sci. 2016, 3, 1500305. 10.1002/advs.201500305 PMC5063141 27774392

[49] F. Liu , Y. Gao , G. Wang , D. Wang , Y. Wang , M. He , X. Ding , H. Duan , S. Luo , Adv. Sci. 2023, 10, 2370019. 10.1002/advs.202204990 PMC9896062 36437047

[50] Y. Xiao , Y. X. Pang , Y. Yan , P. Qian , H. Zhao , S. Manickam , T. Wu , C. H. Pang , Adv. Sci. 2023, 10, 2205292. 10.1002/advs.202205292 PMC10037997 36658693

[51] M. A. Komkova , E. E. Karyakina , A. A. Karyakin , J. Am. Chem. Soc. 2018, 140, 11302. 30118222 10.1021/jacs.8b05223

[52] S. Nie , Z. Li , Y. Yao , Y. Jin , Front. Chem. 2021, 9. 10.3389/fchem.2021.803509 PMC8738075 35004622

[53] J. Li , M. Zhang , J. Liu , Y. Ma , Synth. Met. 2014, 198, 161.

[54] M. Shimomura , M. Kaga , N. Nakayama , S. Miyauchi , Synth. Met. 1995, 69, 313.

[55] L. Shi , H. Huang , L. Sun , Y. Lu , B. Du , Y. Mao , J. Li , Z. Ye , X. Peng , Dalton Trans. 2013, 42, 13265. 23887280 10.1039/c3dt50823d

[56] A. Panwar , M. M. Sk , B. H. Lee , L. P. Tan , RSC Adv. 2022, 12, 7922. 35424739 10.1039/d1ra09084d PMC8982264

[57] S. Farris , J. Song , Q. Huang , J. Agric. Food Chem. 2010, 58, 998. 20043635 10.1021/jf9031603

[58] R. Núñez‐Flores , B. Giménez , F. Fernández‐Martín , M. E. López‐Caballero , M. P. Montero , M. C. Gómez‐Guillén , Food Hydrocoll. 2013, 30, 163.

[59] X. Dang , Z. Shan , H. Chen , Int. J. Biol. Macromol. 2018, 107, 1023. 28939524 10.1016/j.ijbiomac.2017.09.068

[60] L. Guo , T. Qiang , Y. Ma , L. Ren , C. Zhu , ACS Sustainable Chem. Eng. 2021, 9, 8393.

[61] T. Seki , K.‐Y. Chiang , C.‐C. Yu , X. Yu , M. Okuno , J. Hunger , Y. Nagata , M. Bonn , J. Phys. Chem. Lett. 2020, 11, 8459. 32931284 10.1021/acs.jpclett.0c01259 PMC7584361

[62] H.‐J. Shin , S. S. Jeon , S. S. Im , Synth. Met. 2011, 161, 1284.

[63] Z.‐H. Jin , Y.‐L. Liu , J.‐J. Chen , S.‐L. Cai , J.‐Q. Xu , W.‐H. Huang , Anal. Chem. 2017, 89, 2032. 28029034 10.1021/acs.analchem.6b04616

[64] V. N. Nikitina , A. R. Karastsialiova , A. A. Karyakin , Biosens. Bioelectron. 2023, 220, 114851. 36345086 10.1016/j.bios.2022.114851

[65] J.‐Y. Chiu , C.‐M. Yu , M.‐J. Yen , L.‐C. Chen , Biosens. Bioelectron. 2009, 24, 2015. 19042119 10.1016/j.bios.2008.10.010

[66] D. Aradilla , S. Sadki , G. Bidan , Synth. Met. 2019, 247, 131.

[67] D. Ji , X. Guo , W. Fu , Z. Ding , C. Wang , Q. Zhang , S. Ramakrishna , X. Qin , Mater. Sci. Engineer.: R: Rep. 2022, 149, 100681.

[68] B. G. Choi , J. Hong , W. H. Hong , P. T. Hammond , H. Park , ACS Nano 2011, 5, 7205. 21823578 10.1021/nn202020w

[69] R. Kötz , M. Carlen , Electrochim. Acta 2000, 45, 2483.

[70] L. Meng , F. Dagsgård , A. P. F. Turner , W. C. Mak , J. Mater. Chem. C 2020, 8, 12829.

[71] B.‐A. Mei , O. Munteshari , J. Lau , B. Dunn , L. Pilon , J. Phys. Chem. C 2018, 122, 194.

[72] D. Aradilla , M. Delaunay , S. Sadki , J.‐M. Gérard , G. Bidan , J. Mater. Chem. A 2015, 3, 19254.

[73] T. Q. N. Tran , G. Das , H. H. Yoon , Sens. Actuators, B 2017, 243, 78.

[74] S. Uzunçar , L. Meng , A. P. F. Turner , W. C. Mak , Biosens. Bioelectron. 2021, 171, 112725. 33113385 10.1016/j.bios.2020.112725

[75] E. Bakker , ACS Sens. 2016, 1, 838.

[76] W. L. Clarke , D. Cox , L. A. Gonder‐Frederick , W. Carter , S. L. Pohl , Diabetes Care 1987, 10, 622. 3677983 10.2337/diacare.10.5.622

[77] American Diabetes Association Professional Practice Committee , Diabetes Care 2024, 48, S146. 10.2337/dc18-Sdis01 29222387

[78] G. Maroli , G. Rosati , S. Suárez‐García , D. Bedmar‐Romero , R. Kobrin , Á. González‐Laredo , M. Urban , R. Alvárez‐Diduk , D. Ruiz‐Molina , A. Merkoçi , Biosens. Bioelectron. 2024, 260, 116421. 38838572 10.1016/j.bios.2024.116421

[79] S. L. Cowart , M. E. Stachura , Glucosuria, Butterworth Publishers, A Division Of Reed Publishing, Boston, 1990.

[80] Y. Li , Y. Kong , Y. Hu , Y. Li , R. Asrosa , W. Zhang , B. Deka Boruah , A. K. Yetisen , A. Davenport , T.‐C. Lee , B. Li , Lab. Chip. 2024, 24, 2454. 38644805 10.1039/d4lc00163j PMC11060138

[81] K. Derwinska , K. Miecznikowski , R. Koncki , P. J. Kulesza , S. Glab , M. A. Malik , Electroanalysis 2003, 15,1843.

[82] N. O. Gomes , R. T. Paschoalin , S. Bilatto , A. R. Sorigotti , C. S. Farinas , L. H. C. Mattoso , S. A. S. Machado , O. N. J. Oliveira , P. A. Raymundo‐Pereira , ACS Sustainable Chem. Eng. 2023, 11, 2209.

