# Supplementary material for: Prussian‐Blue Catalysis and NFC Synergy: a Battery‐Free Laser‐Induced Graphene‐Based Platform for Urine Glucose Monitoring at Point‐of‐Care
Source: Adv Sci (Weinh). 2025 Mar 6;12(20):2500365. doi: 10.1002/advs.202500365 (PMC12120749; doi:10.1002/advs.202500365)
Supplement: Supplementary file 1 — Supporting Information [file ADVS-12-2500365-s001.docx]

SUPPORTING INFORMATION

**Prussian-Blue Catalysis and NFC Synergy: A Battery-Free Laser-induced Graphene-based Platform for Urine Glucose Monitoring at Point-of-Care**

**Sinan Uzunçar ^1 2^ *, Gabriel Maroli ^1 3^, Urban Massimo ^1 4^, Arben Merkoçi ^1 5^ ***

^1^ Catalan Institute of Nanoscience and Nanotechnology (ICN2), CSIC and BIST, Campus UAB, Bellaterra, 08193 Barcelona, Spain

^2^ Environmental Engineering Department, Engineering Faculty, Zonguldak Bülent Ecevit University, 67100, Zonguldak, Turkey

^3^ Instituto de Investigaciones en Ingeniería Eléctrica Alfredo Desages (IIIE), Universidad Nacional del Sur, Bahía Blanca, Argentina – CONICET

^4^ Facultad de Biociencias, Universitat Autònoma de Barcelona, Campus de la UAB, Bellaterra, Barcelona, 08193, Spain

^5^ Catalan Institution for Research and Advanced Studies (ICREA); Passeig de Lluís Companys, 23, Barcelona, 08010, Spain.

**Corresponding authors*:** [sinan.uzuncar@beun.edu.tr](mailto:sinan.uzuncar@beun.edu.tr)**,** [arben.merkoci@icn2.cat](mailto:arben.merkoci@icn2.cat)

Contents

[1. Experimental 3](#_Toc191775693)

[1.1. Reagents, Apparatus and Measurements 3](#_Toc191775694)

[1.2. Statistical Analysis 3](#_Toc191775695)

[1.3. Synthesis (PEDOT:PB)G-Based PB-CHIA 6](#_Toc191775696)

[1.4. Preparation of Sensors 9](#_Toc191775697)

[*1.4.1.* *GC-Based H_2_O_2_ Sensors* 9](#_Toc191775698)

[*1.4.2.* *GC-Based Glucose Biosensors* 10](#_Toc191775699)

[*1.4.3.* *LIG-Based H_2_O_2_ sensors* 10](#_Toc191775700)

[*1.4.4.* *LIG-Based Glucose Biosensors* 11](#_Toc191775701)

[*1.4.5.* *LIG-Based Fluidic Cell Module Glucose Biosensors* 11](#_Toc191775702)

[2. Results and Discussion 13](#_Toc191775703)

[2.1. Optimization of (PEDOT:PB)G for Glucose Detection on GC 13](#_Toc191775704)

[*2.1.1.* *Optimization of Polymerisation Acidity and Precursor Volume of PB to Generate PB-CHIA* 13](#_Toc191775705)

[*2.1.2.* *Applicability of (PEDOT:PB)G1:4-GOx in Urine-Spiked Medium* 14](#_Toc191775706)

[2.2. Fluidic Cell Module Based on LIG as Nano-Fibrous Fringes (LIG-F) 15](#_Toc191775707)

[*2.2.1.* *Selection of Optimal LIG-based Platform* 15](#_Toc191775708)

[*2.2.2.* *Comparison of GC Electrode and Selected LIG-F-Based Platform* 18](#_Toc191775709)

[*2.2.2.1. Comparison of Bare GC Electrode and LIG-F-Based Platform* 19](#_Toc191775710)

[*2.2.2.1. Comparison of PB-CHIA-Modified GC Electrode and LIG-F-Based Platform* 19](#_Toc191775711)

[2.3. Operational Modes of Fluidic Cell Module-Based Urine-Glucose Sensor 22](#_Toc191775712)

[2.4. NFC System Design and Working Principles 24](#_Toc191775713)

[*2.4.1.* *Design* 24](#_Toc191775714)

[*2.4.2.* *Working Principles* 26](#_Toc191775715)

[3. References 27](#_Toc191775716)

# Experimental

## Reagents, Apparatus and Measurements

3,4-Ethylenedioxythiophene (EDOT), Gelatin powder (Ref Num. 48723), glutaraldehyde (GA, 50%), H_2_O_2_ (30%), FeCl_3_, K_3_[Fe(CN)_6_], K_2_HPO_4_, KH_2_PO_4_, KCl, Glucose Oxidase (GOx) from *Aspergillus niger* (50KU) and glucose were purchased from Sigma-Aldrich and utilized as received. Synthetic urine (Surine™ Negative Urine Control—composition, 95% water, with 9.3 g L^-1^ of urea, 1.87 g L^-1^ of chloride, 1.17 g L^-1^ of sodium, 0.750 g L^-1^ of potassium, 0.670 g L^-1^ of creatinine and other dissolved ions, organic and inorganic compounds from Sigma-Aldrich and stored in the fridge at 4 °C. Milli-Q water (18.2 MΩ) was used to prepare all solutions. Electrochemical measurements, except for electrochemical impedance spectroscopy (EIS), were carried out by Autolab PGSTAT204 instrument (Kanaalweg 29G, 3526 KM Utrecht, Netherlands) connected to a three-electrode system. PalmSens4 electrochemical analyzer was used for EIS. Pt wire, glassy carbon (GC), and Ag/AgCl electrodes were employed as the counter, working, and reference electrodes. Laser engraving was used to obtain the counter, working electrode, and working electrode on the Kapton substrate. The Voltera V-One, a desktop PCB Printertool (Ontario 180 Northfield Dr W, Suite 2 Waterloo, ON N2L 0C7, Canada), was used to draw Ag circuitry lines to ensure the contact of the electrodes with the potentiostat, and reference electrodes were fabricated using Ag/AgCl ink in the same manner. Polyethylene terephthalate (PET, thickness 75 µm) and Dupont grade Kapton HN (polyimide, thickness 75 µm) were purchased from Goodfellow GmbH (Spain). They were laminated with bi-sided tape using a Lamigator IQ (Renz, Spain). A plasma device (Harrick Plasma 110V Model) using 18 W power was employed to control the surface hydrophilicity of the substrates. Three electrodes were utilized for amperometric measurements, each undergoing three measurements for every parameter change. The average of these nine responses, depicted as dots on error bars, was reported as the analytical response, with the error bars representing the standard deviation of these measurements, capturing data variability. In contrast, cyclic voltammetry or impedimetric measurements were obtained using three electrodes, each employed once. The average of the resulting three measurements represents the response, with their standard deviation as error bars.

## Statistical Analysis

In this study, all experimental data were presented as mean values with error bars representing the Standard Deviation (SD) of the datasets shown in the figures. Detailed information for each analysis is as follows:

- **Pre-processing of Data**: No specific data transformation, or outlier evaluation processes were applied. Data were directly used for analysis based on experimental measurements. Normalization was done when reporting the sensitivity and charge density values by dividing raw sensitivity and charge density values by the electrode surface area.
- **Data Presentation**: Charge density values, sensitivity, and LOD (Limit of Detection) were calculated as the mean of multiple measurements. Error bars reflect the SD of these measurements. In the recovery study, three electrodes were fabricated, and the average response from these electrodes was reported. The variability of these responses was represented by the Relative Standard Deviation (RSD).
- **Clarity on Outlier Detection**: In this study, no specific rules for outlier detection were applied to avoid introducing any bias. All experimental data were included in the calculations of mean, SD, and RSD. Due to the nature of the data, some data points may fall outside the error bars derived from the SD.

The equation of the SD is as follows:

SD = $\sqrt{\frac{1}{n-1}\sum_{i=1}^{n} \left( x_{i}-\bar{x} \right)^{2}}$ Equ-1

Where:

- SD = Standard deviation
- n = Number of data points
- x_i_​ = Each individual data point
- $\bar{x}$ = Mean (average) of the data set

The equation of the RSD is as follows:

RSD (%)= $\frac{SD}{\bar{X}}x100$ Equ-2

- **Standard Deviation (SD)** is a measure of the dispersion or spread of the data points from the mean.
- **Mean (Average,** $\bar{x}$**)** is the arithmetic average of the data points.
- **RSD** expresses the standard deviation as a percentage of the mean, indicating variability relative to the average.

1. **Sample Size (n):** Three independent electrodes were fabricated for each experimental condition. Measurements were repeated accordingly (e.g., n=3 for cyclic voltammetry and impedance analysis, n=9 for amperometric measurements).
2. **Statistical Methods:** Statistical analysis focused on direct comparisons of experimental datasets. Mean values and their SDs were calculated. No additional statistical tests were performed beyond these calculations.
3. **Software:** Data processing and graph generation were performed using Origin, Excel, Aftermath, AutoCad and JAPS.

**Presentation of Specific Results:**

- **Charge Density Data:** Raw charge density values were determined by integrating the current with respect to the potential over the redox cycle. The area under the CV-redox curve was divided by the electrode's surface area to obtain the charge density (C/cm²). This value is the average of three datasets with SD error bars. Statistical comparisons between different electrodes in terms of their charge density values were made directly based on the means of these three data points, and p-values were not calculated.

**Formula Used:**

Q_density_ $=\frac{1}{A}\int_{V_{F}}^{V_{L}} I(E) dE$

where:

- - - Q_density_ ​ = Charge density in C/cm²
    - V_F_ and V_L_ = First and the last potentials in V
    - I(E) = Current as a function of potential E in V
    - dE= Differential change in potential in V
    - A = Electrode surface area in cm²
- **Calibration Curve:** Calibration involved three electrodes with analyte additions repeated three times, resulting in nine amperometric measurements for each concentration value. The calibration curves were obtained by plotting the average current values against analyte concentrations. Each point on the graph shown as dots represents the current measured for a specific concentration, and error bars reflect the standard deviation (SD) of these measurements. Sensitivity was determined using linear regression analysis.
- **Sensitivity and LOD Values with Amperometric Analysis:** Raw sensitivity was derived from the slope (μA μM^-1^) of the linear region of the amperometric calibration curve and they were calculated for linear regions with R^2^ > 0.990 to determine the LOD. The dots in the calibration plot correspond to current values at various analyte concentrations, with error bars from SD. Statistical comparisons were conducted using one-way ANOVA and p-values were calculated to assess significant differences between groups using Tukey’s hsd test. The obtained sensitivity values were reported in a common normalized form as μA μM^-1^ cm^-2^.

The LOD was calculated using:

LOD $=\frac{3*SD}{Raw Sensitivity}$

- **Peak Potential, Peak Current, and Impedimetric Analyses:** Three electrodes were fabricated for these analyses. Results are presented as the average of the three analyses. Statistical comparisons between different electrodes in terms of their charge density values were made directly based on the means of these three data points, and p-values were not calculated.

## Synthesis (PEDOT:PB)G-Based PB-CHIA

A solution was prepared to synthesize the PB-infused catalytic hetero-interface architecture (PB-CHIA) by combining 5 mL of 0.1 M HCl + 0.1 M KCl (unless otherwise specified) with 0.5 g of gelatin at 60°C. Next, 160 µL of EDOT, stored in the fridge at +4°C, was added to 340 µL of the gelatin-HCl-KCl solution in a 1 mL Eppendorf tube. The mixture was vigorously vortexed for 1 min until it became milky white. Afterward, 50 µL of the resulting mixture (The remaining part of the mixture can be stored in the fridge at +4°C for further use) was transferred to another Eppendorf tube and combined with varying quantities of 4 M of FeCl_3_ solution (1-2-3-5-10 or 20 µL). This mixture was vortexed forcefully for 1 min until it turned dark green. Lastly, 1-2-3-4 or 5 µL of 1 M K_3_[Fe(CN)_6_] was added to the dark greenish mixture, and it was vortexed again forcefully for 2 min. The mixture's color quickly turned dark blue upon adding K_3_[Fe(CN)_6_], indicating the production of Prussian Blue (PB) nanoparticles.

Simultaneous and sequential reactions form a self-assembling, intertwining PB-CHIA denoted as (PEDOT:PB)G_m_. To tune the thickness of PB-CHIA on the working electrode surface, the dark blue mixture underwent dilution with a 0.1 M HCl + 0.1 M KCl solution at ratios ranging from 1:1 to 1:5. The subscript “m” in (PEDOT:PB)G_m_ [m = a:(a+b)], represents the volumetric ratio of (PEDOT:PB)G without dilution (a) to the total volume (a+b) after the addition of electrolyte (b). For instance, when 150 µL of electrolyte was added to the main polymerization medium (which is always 50 µL), the resulting n value was 1:4 [(50+150)]. Accordingly, the PB-CHIA on the electrode surface is labeled as (PEDOT:PB)G_1:4_. The mixture was vortexed for 5 min, and the reaction temperature was kept at +60°C. To obtain (PEDOT)G without PB, FeCl_3_ is added to EDOT-Gelatin-HCl-KCl without combining it with K_3_[Fe(CN)_6_].

**Figure S1a** depicts the color transformation that ensues when Fe^3+^ and Fe(CN)_6_^3-^ precursors are introduced to the polymerization medium, both with and without EDOT. The gelatine and gelatin-EDOT mixtures were combined with the PB’s precursors after diluting 1:20 with the final volume of 5 mL to make it easier to monitor color change. This control experiment shows that, in both cases, the color change towards blue indicates that both gelatin and EDOT reduce the yielding PB nanoparticles.


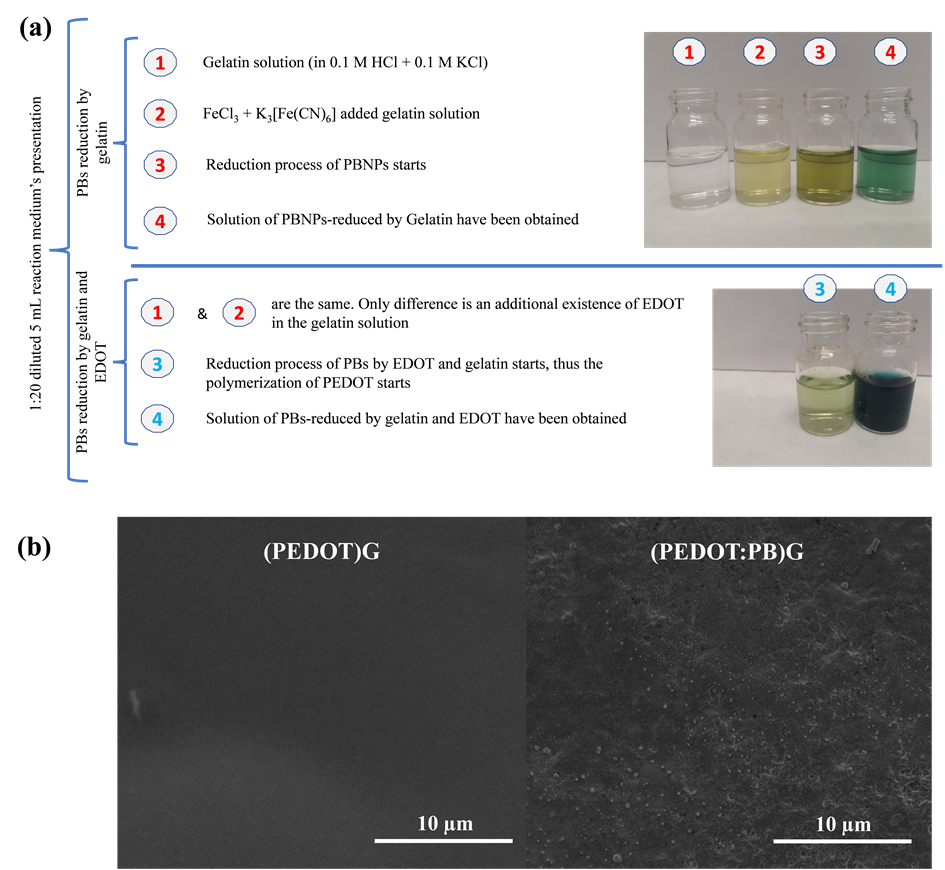


**Figure S1** Evaluation and characterization of the PB-CHIA synthesis. **a)** Effect of EDOT and gelatin on reduction of PB nanoparticles (1:20 diluted mediums), **b)** SEM images of (PEDOT)G and (PEDOT:PB)G interfaces


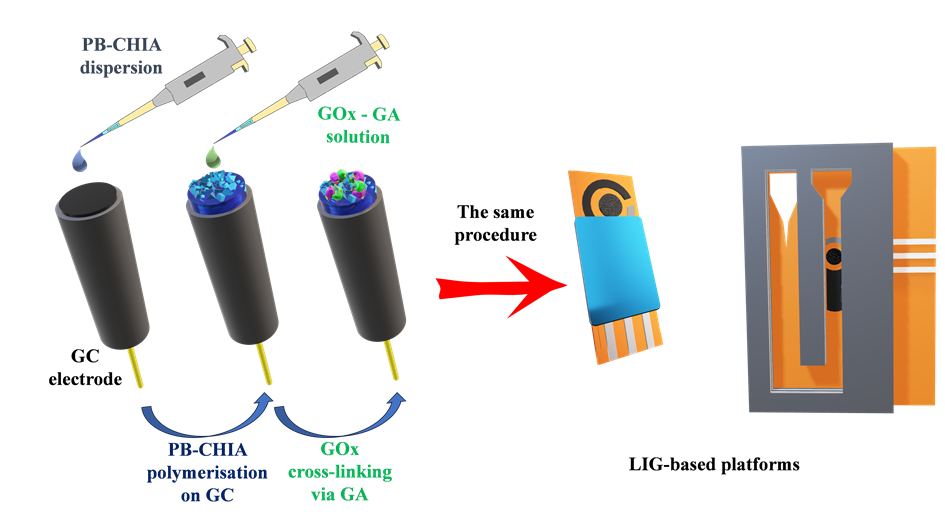


**Figure S2** PB-CHIA and GOx modification of the electrode surfaces. (a) GC and (b) LIG-based electrode modification by drop-casting and cross-linking

## Preparation of Sensors

### *GC-Based H_2_O_2_ Sensors*

In order to prepare the GC-based H_2_O_2_ sensors, GC electrodes (Ø: 3 mm) were first polished with alpha aluminum pastes with particle sizes of 0.3–0.05 µm. They were then washed and sonicated in Milli-Q water for 5 min. These steps were repeated until the redox peaks in the ferric solution were consistent. Once the cleanliness of the electrodes was confirmed, they were further polished again using 0.05 µm diameter aluminum paste, sonicated for an additional 5 min, and subjected to a final wash with Milli-Q water. After these procedures, GC electrodes were ready for modification with (PEDOT:PB)G. As shown in **Figure S2**, 2 µL of the (PEDOT:PB)G dispersion in the polymerization process was dropped on GC electrodes. They were kept under a fume hood for 24 h, in the dark, and subjected to two sequential activation protocols. Firstly, after constructing the three-electrode setup, these electrodes were activated in 0.1 M KCl solution between -0.4/+0.8 V potential window with a scan rate of 50 mV s^-1^ for 25 cycles. Secondly, the surface of the working electrodes was cleaned with 0.05 M PBS + 0.1 M KCl, submerged in a new PBS solution, the three-electrode setup was constructed, and they were cycled between the potential window of -0.2/+0.6 V with a scan rate of 50 mV 50 mV s^-1^ for 25 cycles. Lastly, these electrodes were run in the stirring medium at a constant potential of -0.05 V for 15 min in PBS containing 20 µM H_2_O_2_ to eliminate superficially adsorbed PBs on PB-CHIA films. These electrodes were washed with PBS and immersed in an Eppendorf tube containing PBS and stored at room temperature in the dark until use. Before amperometry analyses, the H_2_O_2_ sensor electrodes were conditioned at room temperature in fresh PBS at a potential of -0.05 V until a steady-state baseline was formed.

### *GC-Based Glucose Biosensors*

Two separate solutions of PBS were prepared to immobilize the enzyme on the PB-CHIA-modified GC electrodes [GC/(PEDOT:PB)G], as explained in section 1.3.1. These solutions contained 8% GA and 28 mg mL^-1^ GOx, respectively. They were combined in equal parts, and 1 µL of the resulting GA-GOx mixture was dropped onto the electrode surface three times, with a 30-minute interval between each application (Figure S2). After the final application of the enzyme solution, the electrode surface was left to dry for 30 minutes. Following this, 1.5 µL of fresh PBS was dropped on the dried surface, and the electrode was stored in a refrigerator at 4°C for 3 hours. The electrodes were then removed from the refrigerator, dipped in PBS at 4°C, and stored with the surface facing upwards for 1 day. After washing the electrode surface with PBS, the electrodes were activated in a new PBS (-0.2/+0.6 V potential window, 50 mV s^-1^ for 25 cycles). After this step, the electrodes were immersed in fresh PBS with the GC surfaces facing downwards and stored in the refrigerator until they were used. Before amperometry analyses, the electrodes were conditioned at room temperature in fresh PBS at a potential of -0.05 V until a steady-state baseline was formed.

### *LIG-Based H_2_O_2_ sensors*

A 40x110 mm polyimide substrate was cleaned with ethanol and placed on the laser printer stand to fabricate several LIG-based sensor platforms, with dimensions shown in **Figure S3a**. The engraving speed was kept constant at 80% for all patterning processes. The working electrode surface (Ø: 3 mm with 2, 7, or 12 mm long safety margins), the counter electrode, and its safety margin (2 mm) were prepared in engraving mode. While the working electrode surface was patterned with varying power levels (30, 45, and 60%), a 30% power level was always used for the safety margins of the working and counter electrodes. Following this, the substrates were subjected to a 2-minute plasma treatment. Subsequently, a PCB printer was used to print the reference electrode and its safety margin (2 mm) using Ag/AgCl ink. Circuit lines connecting each electrode to the potentiostat were then printed using Ag ink. After each ink application, the substrates were heat-treated in an oven at 180°C for 15 minutes. Next, a rectangular-shaped polyline was drawn with dielectric ink (Figure S3b), and the substrate underwent another heat treatment at 180°C for 15 minutes. The cured polyline was manually filled with waterproof varnish, and the substrate was left in the fume hood for one day. Before the PB-CHIA modification of the working electrode surface, a 6-min plasma treatment was applied to all LIG-based platforms, which were then stored in a Petri dish at room temperature until use.

To obtain LIG/(PEDOT:PB)G-based H_2_O_2_ sensors, these platforms were modified using PB-CHIA dispersion, as explained in section 1.3.1. Activation protocols and storage conditions were the same.

### *LIG-Based Glucose Biosensors*

To obtain LIG/(PEDOT:PB)G-GOx-based glucose biosensors, LIG platforms were fabricated and their working electrodes were modified with the PB-CHIA as explained in section 1.3.1. The GOx enzyme immobilization procedure is the same as mentioned in section 1.3.2. Activation protocols and storage conditions were the same.


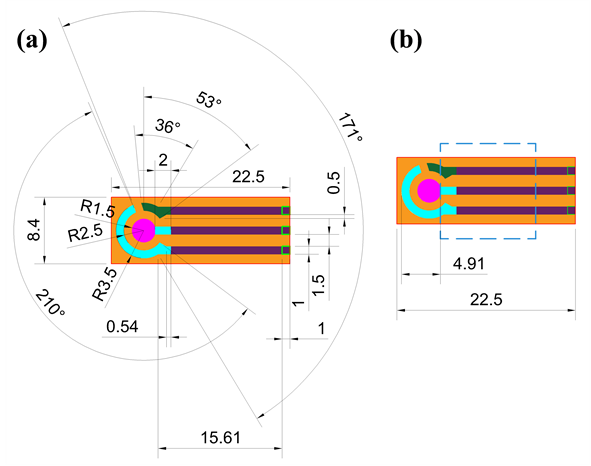


**Figure S3** Dimensions of the three-electrode compartment on Polyimide substrate (LIG-based platforms). **a)** Cutting edges (red), working electrode (pink), counter electrode and neck (cyan), Ag/AgCl ink (dark green), and Ag ink-made tracks (violet). **b)** The dashed blue line indicates the region to be covered with a dielectric and varnish

### *LIG-Based Fluidic Cell Module Glucose Biosensors*

Initially, the layers were constructed according to the dimensions presented in **Figure S4** as part of the fluidic cell fabrication protocol (Step-1). Bi-sided tapes and PET substrates were adhered together and cut using a laser device to create reservoir and flow channel compartments. The three-electrode compartment on polyimide was prepared as described in Section 1.3.3. The designs of the counter and reference electrodes were suited to the fluidic cell's flow channel. Engraving levels and speeds were applied similarly to create the working and counter electrodes with 2 mm safety margins. Subsequently, the flow channel tape was positioned on the polyimide layer without removing the protective layer in contact with the air (Step-2). The assembly then underwent hot lamination 20 times. The polyimide with the flow channel tape-assembled part, still with its protective layer, then underwent a 6-minute plasma treatment (Step-3). Following this, the PB-CHIA modification of the working electrodes of the fluidic cell module was carried out as explained in section 1.3.1, and the GOx enzyme immobilization procedure (Step-4) was performed as in section 1.3.2. After the enzyme immobilization, the PET layer shown in Figure S4e was placed on the fluidic channel patterned multi-layered tape by removing its protective layer. The bi-sided tape was placed on the back side of the three-electrode compartment shown in Figure S4b, and the back side PET coverage of the fluidic cell module (Figure S4a) was put on it. After all layers were placed on the polyimide substrate, the resulting module was cold laminated several times (Step-5). The activation protocol and storage conditions applied to the module were the same as those explained in section 1.3.2. For storage, 150 µL of PBS was added to its inlet, which was then sealed with an easy-to-remove tape, not in contact with the polyimide substrate. The assembled unit was kept in a Petri dish in the fridge before use. Before amperometry analyses, the module was brought to room temperature. Then, after adding 50 µL of fresh PBS from the inlet, it was conditioned at a potential of -0.05 V until a steady-state baseline was formed.


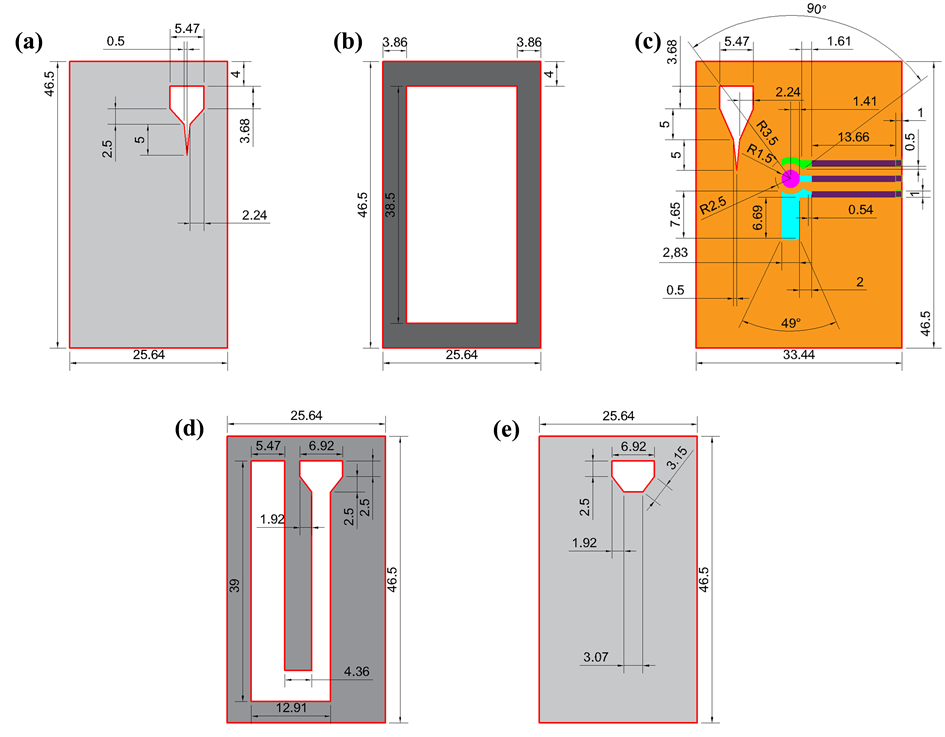


**Figure S4** Compartments and dimensions of the fluidic cell module. **a)** Back layer (PET substrate, light grey), **b)** Reservoir bi-sided tape (dark grey), **c)** Polyimide substrate with inlet cutout, three-electrode system (pink for working electrode, cyan for counter and neck of working electrode, green for Ag/AgCl ink, dark violet for Ag-ink tracks), **d)** Fluidic channel bi-sided tape, **e)** Front layer with inlet cutout (PET substrate). Red lines indicate the cutout edges on the respective substrate

# Results and Discussion

## Optimization of (PEDOT:PB)G for Glucose Detection on GC

### *Optimization of Polymerisation Acidity and Precursor Volume of PB to Generate PB-CHIA*

**Figure S5** presents CV-redox curves of GC electrodes modified with (PEDOT)G (Figure S5a) and (PEDOT:PB)G (Figure S5c) along with the calculated charge density and amperometric sensitivity values (Figure S5b-5d). While (PEDOT)G did not generate a visible amperometric signal, the amperometric sensitivity values for (PEDOT:PB)G remained consistent (oscillated between 220 and 270 µA mM^-1^ cm^-2^) regardless of changes in HCl concentration.

In line with previous reports elsewhere ^[1]^, the presence of KCl in the supporting electrolyte revealed the PB/PW redox peak pair. Following this observation and the literature ^[2–5]^, the polymerization medium’s supporting electrolyte of 0.1 M HCl and 0.1 M KCl during the polymerization process was selected and used to continue the study. To improve the electroanalytical performance of the sensor electrodes, we further investigated the effects of P_1_ and P_2_ volume on the resulting PB-CHIA.


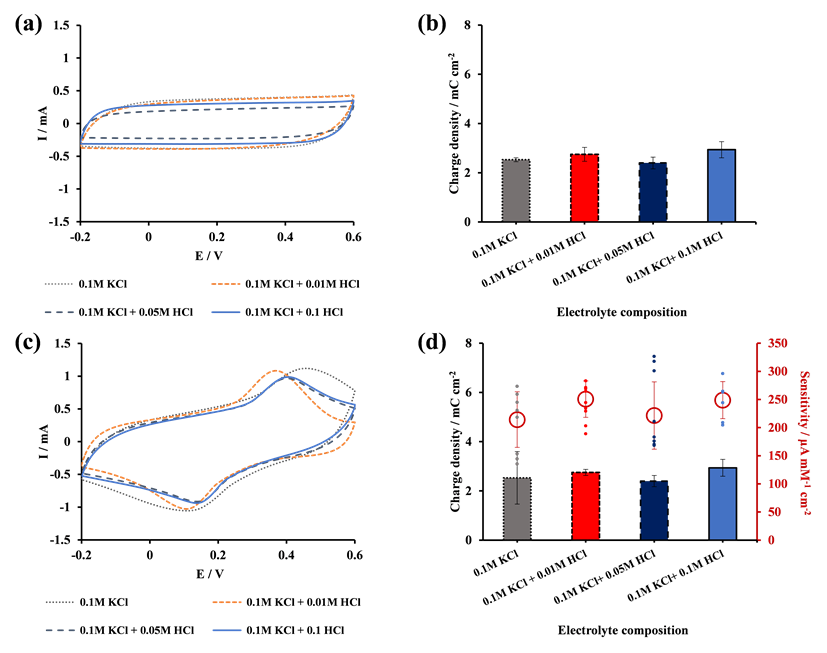


**Figure S5** Electrolyte composition effects of PB-CHIA synthesis. **a)** CV-redox signals and **b)** charge density values of PB-free PB-CHIA synthesized in various supporting electrolytes. **c)** CV-redox signals, **d)** charge density and amperometric sensitivity values of PB-CHIA synthesized in different supporting electrolytes

### *Applicability of (PEDOT:PB)G1:4-GOx in Urine-Spiked Medium*

**Figure S6** Self-life of GC/(PEDOT:PB)G_1:4_-GOx electrodes

**Table S1**. GC/(PEDOT:PB)G_1:4_-GOx electrode’s recovery findings in urine-spiked PBS.

| **Glucose addition (µM)** | **Urine addition (mL)** | **Total volume (mL)** | **Glucose measured (µM)** | **Recovery (%)** | **RSD (%)** |
| --- | --- | --- | --- | --- | --- |
| 7.5 | - | 9.5 | 7.19±0.24 | 95.89±3.16 | 3.29 |
| - | 0.5 | 10 | - | - | - |
| 15 | - | 10 | 14.54±0.42 | 96.92±2.80 | 2.89 |
| 30 | - | 10 | 28.88±1.35 | 96.27±4.51 | 4.68 |
| 45 | - | 10 | 43.05±1.42 | 95.67±3.16 | 3.30 |
| 75 | - | 10 | 70.68±1.26 | 94.25±1.68 | 1.78 |

## Fluidic Cell Module Based on LIG as Nano-Fibrous Fringes (LIG-F)

### *Selection of Optimal LIG-based Platform*

Section 2.2.1 in the manuscript discusses the electrochemical properties of various LIG platforms by investigating their CV-redox, EIS, and amperometric signals, considering both modified and unmodified with PB-CHIA. The compilation of CV-redox peak potentials and currents for bare and PB-CHIA-modified LIGs in ferric and PBS solutions have been presented in **Table S2**-**3** and **Table S4**, respectively.

In the context of the EIS study, it is noteworthy that different circuit models can be constructed using various elements for each electrode. However, conducting a fair comparison becomes challenging in such cases. Therefore, bare and PB-CHIA-modified LIG conditions were distinguished using two separate circuit models (**Figure S7**).


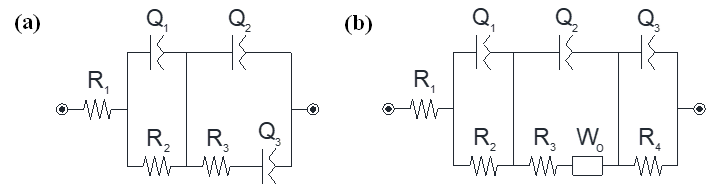


**Figure S7** Equivalent circuit model used to compare different LIGs. **a)** For bare LIGs and **b)** PB-CHIA-modified LIGs operated in PBS

Our primary focus is on the equivalent series resistance (ESR) changes, a critical criterion in EIS studies for comparing sensor platform designs influencing sensor responses. ESR is the internal resistance caused by the bulk electrolyte, electrode, and contact resistance between the electrode and current collector ^[6–9]^, has been denoted as R_1_ in the model.

In addition to the ESR, the constructed EIS models consist of R_2_ solid-electrolyte interface layer resistance, R_3_ charge transfer resistance, R_4_ resistance of double-layer capacitance (C_1_) between the electrode surface coating and liquid, Q_1_, Q_2_, and Q_3_ constant phase elements parallel to these resistance units, and infinite (W) and finite (W_O_) forms of the impedance of Warburg diffusion.

While the bare electrode fits into the "(R_1_s(Q_1_pR_2_)s(Q_2_p(R_3_sQ_3_))" circuit model (Figure S7a) and PB-CHIA-modified LIGs comply with the "(R_1_s(Q_1_pR_2_)s((R_3_sWo_1_)pQ_2_)s(C_1_pR_4_))" circuit model (Figure S7b) in PBS as the electrolyte. The calculated components of EIS circuitry models of bare and PB-CHIA-modified LIG platforms have been gathered in **Table S5** and **Table S6**. In this notation, “s” signifies “in series position” and “p” denotes “in parallel position.

**Table S2**. Bare LIGs with different safety margin lengths and working electrode design: CV-redox data in ferric solution

|  | **E_c_** | **E_a_** | **∆E_a-c_** | **I_c_** | **I_a_** | **∆I_a-c_** |
| --- | --- | --- | --- | --- | --- | --- |
| **Bare** | V | V | V | mA | mA | mA |
| **2-LIG_30%_** | 0.233 | 0.114 | 0.119 | 0.071 | -0.098 | 0.149 |
| **2-LIG_45%_** | 0.266 | 0.092 | 0.174 | 0.094 | -0.091 | 0.185 |
| **2-LIG_60%_** | 0.237 | 0.129 | 0.108 | 0.114 | -0.118 | 0.232 |
| **7-LIG_60%_** | 0.466 | -0.102 | 0.568 | 0.067 | -0.064 | 0.131 |
| **12-LIG_60%_** | 0.805 | -0.474 | 1.279 | 0.048 | -0.051 | 0.099 |

**Table S3**. (PEDOT:PB)G_1:4_-modified LIG with different safety margin lengths and working electrode design: CV-redox data in ferric solution

|  | **E_c_** | **E_a_** | **∆E_a-c_** | **I_c_** | **I_a_** | **∆I_a-c_** |
| --- | --- | --- | --- | --- | --- | --- |
| **Modified** | V | V | V | mA | mA | mA |
| **2-LIG_30%_** | -0.015 | 0.374 | 0.389 | -0.210 | 0.208 | 0.418 |
| **2-LIG_45%_** | 0.002 | 0.307 | 0.305 | -0.340 | 0.312 | 0.652 |
| **2-LIG_60%_** | -0.008 | 0.326 | 0.334 | -0.338 | 0.357 | 0.695 |
| **7-LIG_60%_** | -0.469 | 0.743 | 1.212 | -0.141 | 0.129 | 0.27 |
| **12-LIG_60%_** | -0.442 | 0.913 | 1.355 | -0.047 | 0.088 | 0.135 |

**Table S4**. (PEDOT:PB)G_1:4_-modified LIG with different safety margin lengths and working electrode design: CV-redox data in PBS solution

|  | **E_c_** | **E_a_** | **∆E_a-c_** | **I_c_** | **I_a_** | **∆I_a-c_** |
| --- | --- | --- | --- | --- | --- | --- |
| **Modified** | V | V | V | mA | mA | mA |
| **2-LIG_30%_** | -0.151 | 0.522 | 0.673 | -0.312 | 0.366 | 0.678 |
| **2-LIG_45%_** | -0.058 | 0.311 | 0.369 | -0.469 | 0.512 | 0.981 |
| **2-LIG_60%_** | -0.117 | 0.399 | 0.516 | -0.523 | 0.555 | 1.078 |
| **7-LIG_60%_** | - | - | - | - | - | - |
| **12-LIG_60%_** | - | - | - | - | - | - |

**Table S5**. Bare LIG platforms with different safety margin lengths and working electrode design: EIS data in PBS solution

|  | **R_1_** | **R_2_** | **R_3_** | **R_4_** | **Q_1_** | **α** | **Q_2_** | **α** | **Q_3_** | **α** | **C_dl_** | **Wo** | **B** |
| --- | --- | --- | --- | --- | --- | --- | --- | --- | --- | --- | --- | --- | --- |
| **Bare……..** | kΩ | kΩ | kΩ | kΩ | sᵅ/kΩ |  | sᵅ/kΩ |  | sᵅ/kΩ |  | mF | kΩ/√s | δ/√D |
| **2-LIG_30%_** | 0.691 | 0.074 | 0.061 | - | 0.051 | 0.688 | 0.010 | 0.730 | 0.040 | 0.942 | - | - | - |
| **2-LIG_45%_** | 0.526 | 0.014 | 0.019 | - | 0.116 | 0.802 | 0.025 | 0.639 | 0.069 | 0.985 | - | - | - |
| **2-LIG_60%_** | 0.324 | 0.061 | 0.023 | - | 0.203 | 0.527 | 0.068 | 0.908 | 0.072 | 0.869 | - | - | - |
| **7-LIG_60%_** | 3.237 | 0.014 | 0.222 | - | 0.028 | 0.833 | 0.031 | 0.580 | 0.118 | 1.000 | - | - | - |
| **12-LIG_60%_** | 8.331 | 0.076 | 0.109 | - | 0.044 | 1.000 | 0.001 | 0.853 | 0.173 | 0.939 | - | - | - |

**Table S6**. (PEDOT:PB)G_1:4_-modified LIG platforms with different safety margin lengths and working electrode design: EIS data in PBS solution

|  | **R_1_** | **R_2_** | **R_3_** | **R_4_** | **Q_1_** | **α** | **Q_2_** | **α** | **Q_3_** | **α** | **C_dl_** | **W_o_** | **B** |
| --- | --- | --- | --- | --- | --- | --- | --- | --- | --- | --- | --- | --- | --- |
| **Modified…** | kΩ | kΩ | kΩ | kΩ | sᵅ/kΩ |  | sᵅ/kΩ |  | sᵅ/kΩ |  | mF | kΩ/√s | δ/√D |
| **2-LIG_30%_** | 0.504 | 0.234 | 0.116 | 0.027 | 0.013 | 0.948 | 0.145 | 0.522 | - | - | 0.018 | 0.230 | 1.039 |
| **2-LIG_45%_** | 0.343 | 0.065 | 0.046 | 0.020 | 3.897 | 0.538 | 0.065 | 0.722 | - | - | 6.559 | 0.027 | 0.167 |
| **2-LIG_60%_** | 0.266 | 0.036 | 0.054 | 0.024 | 8.175 | 0.803 | 0.020 | 0.709 | - | - | 53.548 | 0.075 | 0.587 |
| **7-LIG_60%_** | 3.434 | 0.187 | 0.191 | 0.038 | 5.046 | 0.646 | 0.705 | 0.549 | - | - | 0.000 | 0.040 | 0.152 |
| **12-LIG_60%_** | 7.052 | 0.131 | 0.026 | 0.039 | 1.002 | 0.686 | 0.968 | 0.749 | - | - | 0.001 | 0.020 | 0.074 |

### *Comparison of GC Electrode and Selected LIG-F-Based Platform*

After selecting the optimal LIG platform (2-LIG_60%_, hereafter referred to as LIG-F), differences between the GC electrode and the chosen platform were identified through a detailed analysis of CV-redox and EIS. CV-redox signal variations are also evident for the bare electrodes in **Figure S8b** (operated in PBS) and Figure S8c (in ferric solution), as well as PB-CHIA-modified electrodes in Figure S8d (in PBS) and Figure S8e (in ferric solution). The EIS study exclusively utilized ferric solution as the electrolyte.

The bare GC electrode and LIG-based platform conform to the "(R_1_s((R_2_sW_1_)pQ_1_))" and "(R_1_s((R_2_sWo_1_)pQ_1_))" simplified Randles model, as seen in Figure S8a. CV-redox peak potentials and current data have been compiled in **Table S7**-**9**-**10**, while EIS study data has been presented in **Table S8**.

### *2.2.2.1. Comparison of Bare GC Electrode and LIG-F-Based Platform*

Figure S8 highlights the CV-redox and EIS analysis signals comparing the bare-GC and the LIG-F platform in both PBS and ferric solutions. In PBS, as expected, the LIG-F platform produces box-shaped CV-redox signals (Figure S8b) due to the existence of graphene structures atypical of the GC electrode. The LIG-F platform exhibits a higher charge density value (for LIG-F: 38.708x10^-3^ mC cm^-2^, for GC: 0.027x10^-3^ mC cm^-2^). In the ferric solution (Figure S8c and Table S8), the LIG-F platform has a higher ∆I_a-c_ value, while its ∆E_a-c_ value is larger than that of the GC electrode due to additional resistances introduced by the safety margin (for 2-LIG_60%_ ∆E_a-c_: 0.382 V, ∆I_a-c_: 0.598 mA, for GC ∆E_a-c_: 0.312 V, ∆I_a-c_: 0.430 mA).

The EIS signals from the GC electrode conform to a Randles model in the ferric solution. This model includes a constant phase element (Q_1_) in parallel with the series combination of R_2_ (charge transfer resistance) and W, as well as an R_1_ (ESR) that is connected in series with the entire system. Regarding the 2-LIG_60%_ electrode, Q_2_ is included in the system connected in series, and Wo replaces W. The calculated ESR value of 2-LIG_60%_ is higher (0.263 kΩ) than on the GC electrode (0.095 kΩ) because of the additional resistivity coming from the safety margin, and the charge transfer resistance value remains relatively lower (0.075 kΩ) than on the GC electrode has 0.434 kΩ (Table S10).

### *2.2.2.1. Comparison of PB-CHIA-Modified GC Electrode and LIG-F-Based Platform*

The electrochemical results show a similar parameter trend when PB-CHIA modifies the GC and LIG-F electrodes, as presented in Table S9-10. The PB-CHIA-modified LIG-F platform's ∆I_PB-PW_ (1.948 mA) value is higher than that of the PB-CHIA-modified GC electrode (1.516 mA). The PB-CHIA-modified GC’s ∆E_PB-PW_ value (0.653 V) is lower than that of the PB-CHIA-modified LIG-F electrode (1.024 V) due to additional resistances from the electrical transmission line, including the safety margin. It is seen that there is no significant difference in charge density values (for GC/(PEDOT:PB)G and LIG-F/(PEDOT:PB)G it was 1.300±0.080 mC cm^-2^ and 1.222±0.111 mC cm^-2^, respectively). Still, when the sensitivity values are considered, there is a considerable difference when switching from the GC to the LIG-F electrode within the same linear working region of 10-160 µM H_2_O_2_. Whereas GC/(PEDOT:PB)G provides a sensitivity of 304.401 µA mM^-1^ cm^-2^, the LIG-F/(PEDOT:PB)G platform achieves to be 433.332 µA mM^-1^ cm^-2^. Lastly, although the sensitivity value of the LIG electrode remains low (25.39±2.98 μA mM^-1^ cm^-2^) compared to GC (48.62±3.42 μA mM^-1^ cm^-2^) after PB-CHIA and GOx modification which might be a side effect of enzyme immobilization process, the LIG electrode offers the opportunity to work in a broader (10-600 µM compared to 10-160 µM glucose) linear working region, validating that LIGs increase the contact surface area between the electrode and film and yield interfacial porosity enhancing the charge transfer dynamics between the solid-to-liquid junction, improving electroanalytical performance in terms of the linear working range.


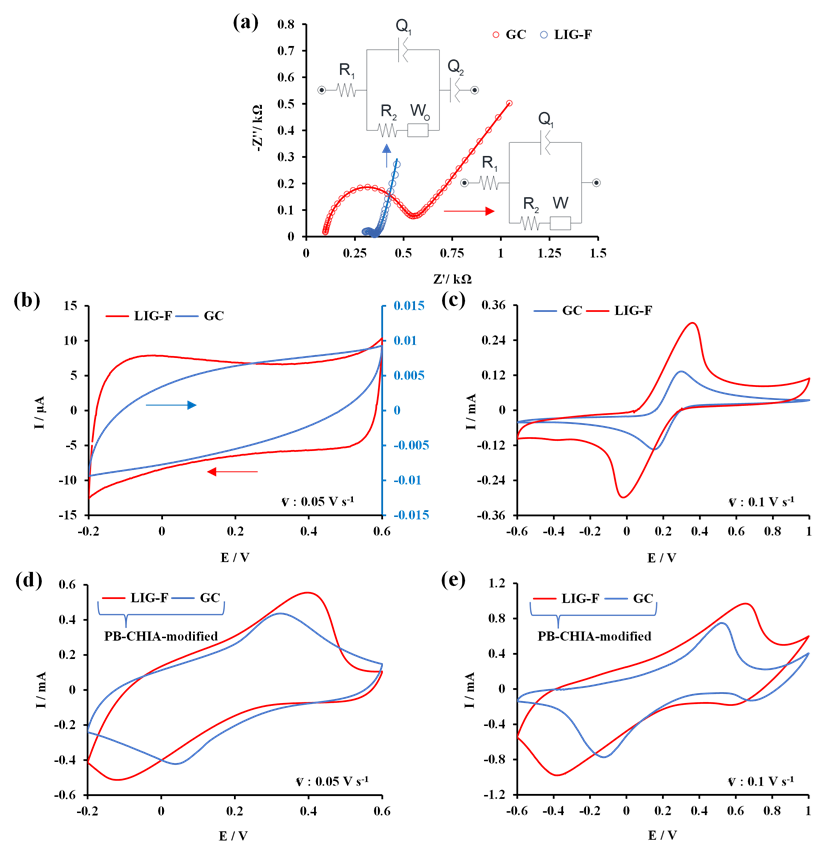


**Figure S8** Electrochemical comparison of GC and LIG-F bare electrodes. **a)** EIS signals of GC and LIG-F electrodes in ferric (between 1x10^3^ and 0.1 Hz, E_dc_ =0.2 V E_ac_ = 0.005 V, equilibration time = 5 sec). **b)** CV-redox signals of bare GC and LIG-F electrodes in PBS and **c)** ferric solution. **d)** CV-redox signals of PB-CHIA-modified GC and LIG-F electrodes in PBS and **e** ferric solution

| 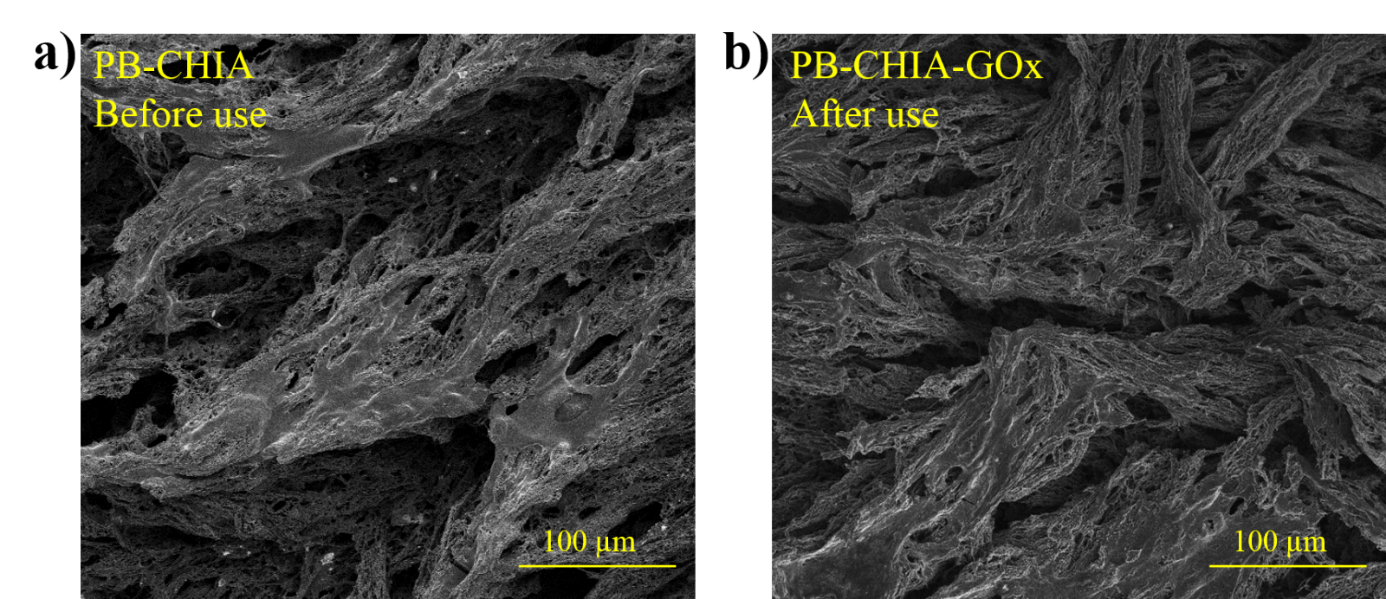 |
| --- |
| **Figure S9.** SEM images of the PB-CHIA and PB-CHIA-GOx, **a)** as deposited on the LIG electrodes and **b)** after cross-linking with GA in the presence of GOx, and use (>30 amperometric measurements) |

**Table S7**. Bare GC electrode and LIG-F platform: CV-redox data in ferric solution (ⱴ: 0.1 V s^-1^)

|  | **E_c_** | **E_a_** | **∆E_a-c_** | **I_c_** | **I_a_** | **∆I_a-c_** |
| --- | --- | --- | --- | --- | --- | --- |
| **Bare** | V | V | V | mA | mA | mA |
| **GC** | -0.012 | 0.300 | 0.312 | -0.298 | 0.132 | 0.430 |
| **LIG-F** | -0.016 | 0.366 | 0.382 | -0.298 | 0.300 | 0.598 |

**Table S8**. Bare GC electrode and LIG-F platform: EIS data in ferric solution

|  | **R_1_** | **R_2_** | **Q_1_** | **α** | **Q_2_** | **α** | **Q_2_** | **α** | **W** | **B** | **Wo** | **B** | **-** |
| --- | --- | --- | --- | --- | --- | --- | --- | --- | --- | --- | --- | --- | --- |
| **Modified** | kΩ | kΩ | sᵅ/kΩ |  | sᵅ/kΩ |  | sᵅ/kΩ |  | kΩ/√s | δ/√D | kΩ/√s | δ/√D | - |
| **GC** | 0.095 | 0.434 | 0.003 | 0.896 | - | - | - | - | 0.569 | - | - | - | - |
| **LIG-F** | 0.263 | 0.075 | 0.030 | 0.619 | 10.791 | 0.598 | - | - | - | - | 0.063 | 0.771 | - |

**Table S9**. PB-CHIA-modified GC electrode and LIG-F platform: CV-redox data in PBS solution (ⱴ: 0.1 V s^-1^)

|  | **E_c_** | **E_a_** | **∆E_a-c_** | **I_c_** | **I_a_** | **∆I_a-c_** |
| --- | --- | --- | --- | --- | --- | --- |
| **Modified** | V | V | V | mA | mA | mA |
| **GC** | 0.042 | 0.323 | 0.365 | -0.422 | 0.437 | 0.859 |
| **LIG-F** | -0.121 | 0.402 | 0.523 | -0.512 | 0.555 | 1.067 |

**Table S10**. PB-CHIA-modified GC electrode and LIG-F platform: CV-redox data in ferric solution (ⱴ: 0.1 V s^-1^)

|  | **E_c_** | **E_a_** | **∆E_a-c_** | **I_c_** | **I_a_** | **∆I_a-c_** |
| --- | --- | --- | --- | --- | --- | --- |
| **Modified** | V | V | V | mA | mA | mA |
| **GC** | -0.121 | 0.532 | 0.653 | -0.774 | 0.742 | 1.516 |
| **LIG-F** | -0.379 | 0.645 | 1.024 | -0.980 | 0.968 | 1.948 |

|  |
| --- |
| **Figure S10** Selectivity and interference study using model urine with the LIG-F-based fluidic cell sensor: The bar graph shows the responses of individual interfering substances and 25 µM glucose, normalized to the 100 µM glucose reading. The inset presents amperometric readings from the interference study, demonstrating the addition of 25 µM glucose and 25 µM glucose with interfering substances (n=3). |

## Operational Modes of Fluidic Cell Module-Based Urine-Glucose Sensor

The sensor operates in two modes: limited batch-flow injection (**Figure S11a**) and limitless batch-flow injection (Figure S11b). In the former mode, samples can be added until the flow channel and reservoir reach a capacity of 400 µL. The sensor must be positioned parallel to the ground for the batch mode. In the latter mode, the sensor is tilted at a 75° angle with a napkin placed at the back of the platform, which allows for the addition of limitless samples.

Both modes follow a common principle, and their usage is as follows:

1. Sample additions to the sensor should be around 50 µL, replacing the former samples in the three-electrode compartment.

2. Before adding a synthetic urine sample diluted 1:20 with PBS, a blank PBS is added to establish a new reference point consistently.

3. Blank PBS is added to initiate device operation. Amperometry begins by applying a -0.05 V potential; at least 200 sec is needed for the current values to stabilize.

4. A synthetic urine sample diluted 1:20 with PBS is added, and the peak reduction current is obtained within 15 seconds.

5. The difference between this peak reduction current value and the previous steady state current (µA) is utilized (as indicated in the main text, Section 2.3) in the calibration equation to determine the sample's glucose concentration (µM).

6. If a new urine glucose reading is required immediately after, at least 200 sec is needed to wait for the amperometric current values to stabilize again.

7. A new blank PBS is added, and another 200 seconds are waited before repeating steps 4 and 5.

8. A blank PBS is added to clean the three-electrode compartment electrochemically before turning off the device. A wait of at least 200 seconds precedes terminating amperometry.

For sensor storage:

1. After step 8, the fluidic cell module is placed on a fresh and dry napkin, and 1 mL of blank PBS, which the napkin absorbs, is slowly added to the inlet part of the device.

2. The sensor is gently shaken to remove as much liquid as possible.

3. For an extended shelf life, 150 µL blank PBS can be added, the inlet section closed, and the sensor stored at +4° degrees.


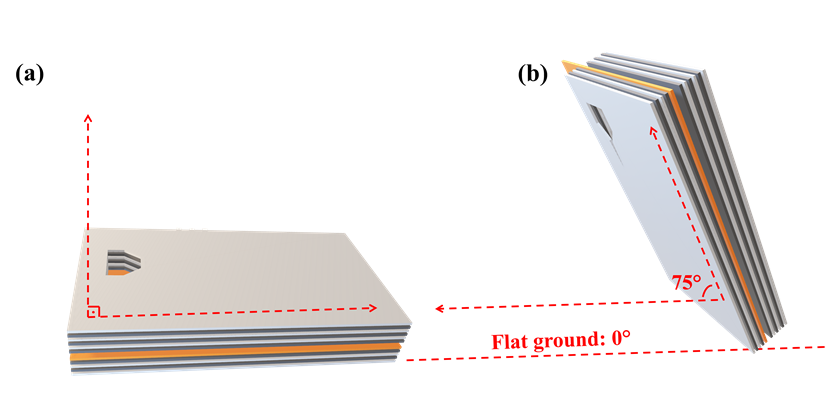


**Figure S11** Operation modes of fluidic cell module. **a** Limited batch-flow-injection **b** Limitless batch-flow-injection

**Table S11**. Recovery values of LIG-F_FC_/(PEDOT:PB)G_1:4_-GOx sensor using artificial urine diluted with PBS.

|  | **With 1:20 diluted synthetic urine** | | |
| --- | --- | --- | --- |
| **50 µL Sample added** | **Glucose measured** | **Recovery** | **RSD** |
| µM | µM | % | % |
| 30 | 31.99±1.28 | 106.62±4.25 | 4.25 |
| 60 | 54.48±2.12 | 90.81±3.53 | 3.89 |
| 100 | 100.68±1.12 | 100.68±1.12 | 1.12 |

## NFC System Design and Working Principles

### *Design*

For the pursuit of advanced portable electrochemical reading, we employed a potentiostat embedded within a single chip that possesses both power supply and NFC transmission capabilities. This state-of-the-art technology was specifically chosen to design a point-of-care device that eschews the need for additional batteries. Central to our design is the SIC4341 chip, a pioneering product of the Thai-based company Silicon Craft Technology. This chip allows many electrochemical techniques such as cyclovoltammetry (CV), Square Wave Voltammetry (SWV), difference pulse voltammetry (DPV), among others, applying voltages from -0.8V to 0.8V. But our main interest lies in the capability to perform chronoamperometry for several hours and the possibility to choose between 2 current ranges, 2.5x10^-3^ mA and 20 x10^-3^ mA, which makes this chip perfect for the present application, as seen in the video of the supplementary information. Instead of employing the manufacturer's demo board, we ventured to craft a custom flexible antenna. This move was imperative to achieve our ultimate goal of device miniaturization.

The NFC technology predominantly operates at a frequency of 13.56MHz. Designing an antenna that resonates at this frequency requires meticulous attention to the chip's input capacitance. Gleaning from the manufacturer's datasheet, the input capacitance of the SIC4341 chip is noted to be 51 pF. Given the RLC circuit under consideration (as depicted in Lumped Model ^[10]^), the necessary inductance, L, can be ascertained through the equation $f_{0}= \frac{1}{2\pi\sqrt{L {(C}_{Chip}+C_{parasite})}}$. Here $f_{0}=13.56MHz$ is the resonance frequency, C_chip_= 51pF represents the chip's input capacitance, C_parasite_ denotes the antenna's parasitic capacitance, and L marks the required inductance for our antenna. A refined inductance value requires an iterative process. Initial calculations, ignoring the parasitic capacitance, furnished a seed inductance value of 2.7 μH. The design realm for planar antennas is replete with various models, notably the Modified Wheeler Formula, Expression Based on Current Sheet Approximation, and the Data Fitted Monomial Expression. Each model demands unique geometrical adjustments. Our primary antenna design comprised a square coil with an external diameter, D_out,_ of 22.987 μm (905 mil), trace width, w, of 381 μm (15 mil), trace separation, s, of 203 μm (8 mil), a number of turns, N, of 10, and resulting in a theoretical inductance, L, of 2.69 μH. Post calculation of the parasitic capacitance, we revised the inductance value. Our finalized design featured a square inductor with D_out_ = 20.320 μm (800 mil), w = 381 μm (15 mil), s = 203 μm (8 mil), with 10 turns, leading to a theoretical inductance, L, of 2.1 μH. While a reduced inductor size might appear advantageous, the diminished inductance impairs the antenna's quality factor (Q). It is important to understand that Q links radiated power to power loss, articulated as 𝑄=(𝑅𝑎𝑑𝑖𝑎𝑡𝑒𝑑 𝑃𝑜𝑤𝑒𝑟)/(𝑃𝑜𝑤𝑒𝑟 𝐿𝑜𝑠𝑠), or alternatively, 𝑄=(𝐼𝑛𝑑𝑢𝑐𝑡𝑖𝑣𝑒 𝑅𝑒𝑎𝑐𝑡𝑎𝑛𝑐𝑒)/𝑅𝑒𝑠𝑖𝑠𝑡𝑎𝑛𝑐𝑒, 𝑄=ω𝐿/𝑅 =2π𝑓𝐿/𝑅. As deduced from this formula, a decline in inductance directly compromises the quality factor. The antenna's final design was meticulously developed using Altium Designer Summer 09 and was fabricated by PCBWAY in China. The chosen specifications were a Polyimide Flex substrate with a thickness of 150 μm and copper traces of 55 μm in thickness. Both the design and the resultant antenna can be observed in **Figure 5h** (main text). The antenna was fabricated on polyimide, followed by soldering the chip onto it, demonstrating the feasibility of a fully integrated system on a single polyimide substrate. The separation of the Antenna and chip system was intentionally chosen to facilitate various tests.

### *Working Principles*

The fluidic cell employs the SIC4341 chip developed by Silicon Craft. For the power supply and information transmission, the chip receives an amplitude-modulated (AM) signal via the antenna. The information is transmitted at the fundamental frequency of the wave, 13.56 MHz, while its amplitude is modulated by the information.

Upon receiving the signal, the chip’s demodulator extracts the envelope of the signal, which contains the information. To power the chip, the signal is rectified by a diode bridge, transforming the sinusoidal signal into a lobed signal. This signal charges a capacitor that smooths the voltage. To obtain a pure direct current, a Low Drop-Out regulator (LDO) is used. The resulting voltage powers the chip’s logic and facilitates measurements.

In addition, the SIC4341 chip incorporates an “electrochemical interface”. This interface is an array of operational amplifiers, which allows us to perform chronoamperometry and thus measure the electrical signal.


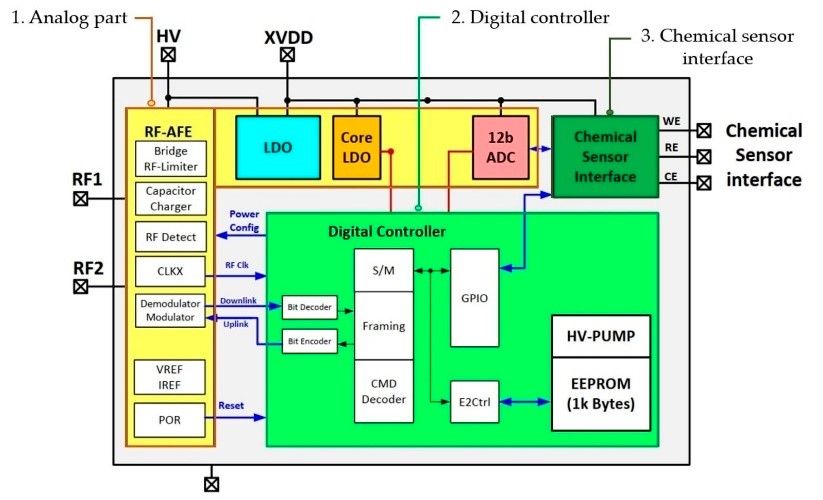


**Figure S12** The schematic of the SIC 4341, as reported in ^[11]^, depicts the analog section where the information is extracted and energy is harvested, as well as the digital block, where the system logic is located, and the electrochemical interface (Copyright © 2021 by the authors. Licensee MDPI, Basel, Switzerland)

# References

[1] S. Uzunçar, H. Kaç, M. Ak, *Talanta* **2023**, *252*, 123841.

[2] A. A. Karyakin, E. E. Karyakina, L. Gorton, *Electrochem commun* **1999**, *1*, 78.

[3] M. A. Komkova, E. E. Karyakina, A. A. Karyakin, *J Am Chem Soc* **2018**, *140*, 11302.

[4] M. A. Komkova, K. R. Vetoshev, E. A. Andreev, A. A. Karyakin, *Dalton Trans.* **2021**, *50*, 11385.

[5] V. N. Nikitina, A. R. Karastsialiova, A. A. Karyakin, *Biosens Bioelectron* **2023**, *220*, 114851.

[6] B. G. Choi, J. Hong, W. H. Hong, P. T. Hammond, H. Park, *ACS Nano* **2011**, *5*, 7205.

[7] R. Kötz, M. Carlen, *Electrochim Acta* **2000**, *45*, 2483.

[8] L. Meng, F. Dagsgård, A. P. F. Turner, W. C. Mak, *J. Mater. Chem. C* **2020**, *8*, 12829.

[9] B.-A. Mei, O. Munteshari, J. Lau, B. Dunn, L. Pilon, *The Journal of Physical Chemistry C* **2018**, *122*, 194.

[10] G. Maroli, A. Fontana, S. M. Pazos, F. Palumbo, P. Julián, in *2021 Argentine Conference on Electronics (CAE)*, **2021**, pp. 61–66.

[11] K. Krorakai, S. Klangphukhiew, S. Kulchat, R. Patramanon, *Applied Sciences* **2021**, *11*, DOI 10.3390/app11010392.
